# Immunophenotypic and Ultrastructural Analysis of Mast Cells in Hermansky-Pudlak Syndrome Type-1: A Possible Connection to Pulmonary Fibrosis

**DOI:** 10.1371/journal.pone.0159177

**Published:** 2016-07-26

**Authors:** Arnold S. Kirshenbaum, Glenn Cruse, Avanti Desai, Geethani Bandara, Maarten Leerkes, Chyi-Chia R. Lee, Elizabeth R. Fischer, Kevin J. O’Brien, Bernadette R. Gochuico, Kelly Stone, William A. Gahl, Dean D. Metcalfe

**Affiliations:** 1 Laboratory of Allergic Diseases, National Institute of Allergy and Infectious Diseases, National Institutes of Health, Bethesda, Maryland, United States of America; 2 Bioinformatics and Computational Biosciences Branch, National Institute of Allergy and Infectious Diseases, National Institutes of Health, Bethesda, Maryland, United States of America; 3 Laboratory of Pathology, National Cancer Institute, National Institutes of Health, Bethesda, Maryland, United States of America; 4 Research Technologies Branch, National Institute of Allergy and Infectious Diseases, National Institutes of Health, Bethesda, Maryland, United States of America; 5 Office of the Clinical Director, National Human Genome Research Institute, National Institutes of Health, Bethesda, Maryland, United States of America; 6 Medical Genetics Branch, National Human Genome Research Institute, National Institutes of Health, Bethesda, Maryland, United States of America; University of Bari Medical School, ITALY

## Abstract

Hermansky-Pudlak Syndrome type-1 (HPS-1) is an autosomal recessive disorder caused by mutations in *HPS1* which result in reduced expression of the HPS-1 protein, defective lysosome-related organelle (LRO) transport and absence of platelet delta granules. Patients with HPS-1 exhibit oculocutaneous albinism, colitis, bleeding and pulmonary fibrosis postulated to result from a dysregulated immune response. The effect of the *HPS1* mutation on human mast cells (HuMCs) is unknown. Since HuMC granules classify as LROs along with platelet granules and melanosomes, we set out to determine if HPS-1 cutaneous and CD34+ culture-derived HuMCs have distinct granular and cellular characteristics. Cutaneous and cultured CD34+-derived HuMCs from HPS-1 patients were compared with normal cutaneous and control HuMCs, respectively, for any morphological and functional differences. One cytokine-independent HPS-1 culture was expanded, cloned, designated the HP proMastocyte (HPM) cell line and characterized. HPS-1 and idiopathic pulmonary fibrosis (IPF) alveolar interstitium showed numerous HuMCs; HPS-1 dermal mast cells exhibited abnormal granules when compared to healthy controls. HPS-1 HuMCs showed increased CD63, CD203c and reduced mediator release following FcɛRI aggregation when compared with normal HuMCs. HPM cells also had the duplication defect, expressed FcɛRI and intracytoplasmic proteases and exhibited less mediator release following FcɛRI aggregation. HPM cells constitutively released IL-6, which was elevated in patients’ serum, in addition to IL-8, fibronectin-1 (FN-1) and galectin-3 (LGALS3). Transduction with *HPS1* rescued the abnormal HPM morphology, cytokine and matrix secretion. Microarray analysis of HPS-1 HuMCs and non-transduced HPM cells confirmed upregulation of differentially expressed genes involved in fibrogenesis and degranulation. Cultured HPS-1 HuMCs appear activated as evidenced by surface activation marker expression, a decrease in mediator content and impaired releasibility. The near-normalization of constitutive cytokine and matrix release following rescue by *HPS1* transduction of HPM cells suggests that HPS-1 HuMCs may contribute to pulmonary fibrosis and constitute a target for therapeutic intervention.

## Introduction

Hermansky-Pudlak syndrome (HPS) is a multisystem disorder that is inherited in an autosomal recessive manner and is characterized by tyrosinase-positive oculocutaneous albinism and a bleeding diathesis resulting from a platelet storage pool deficiency and absence of platelet dense bodies [1]. Of the 9 genetic subtypes of HPS, types 1, 2, and 4 are also associated with a progressive, fatal pulmonary fibrosis typically affecting middle-aged individuals [2,3]. Human mast cells (HuMCs) are terminally differentiated, tissue-residing cells that develop from bone marrow CD34+/CD117+ hematopoietic progenitor cells [4] and have a central role in atopic disorders. HuMCs are found in multiple organs, generally localize to sites interfacing with the external environment, and are involved in homeostasis and pathologic diseases, including fibrosis [5,6]. Since human mast cell granules, along with platelet granules and melanosomes [7] classify as lysosome related organelles (LROs), we set out to examine the mast cell compartment in patients with HPS-1. As will be shown, studies of primary HPS-1 HuMCs revealed that HPS1 protein is involved with granular ultrastructure, expression of surface markers and degranulation. Furthermore, we derived a stable Hermansky-Pudlak proMastocyte (HPM) single cell-cloned line that contains an *HPS1* mutation. This model of primary HPS-1 HuMCs constitutively released cytokines and extracellular matrix proteins.

## Materials and Methods

### Patients and Healthy Subjects

Six HPS-1 patients and five healthy controls were studied following informed consent under NIAID protocols 10-I-0148 and 98-I-0027 and NHGRI protocol 95-HG-0193. All HPS-1 patients and healthy controls underwent skin biopsies, and three from each group had leukapheresis for CD34+ cell enrichment. All HPS-1 individuals were homozygous for the northwest Puerto Rican founder mutation, a 16-bp duplication (c.1470_1486dup16) in the *HPS1* gene, which is the best characterized type of the disease [8]. As shown in Table 1, patients with HPS-1 were adult males age 24–47; one had moderate pulmonary fibrosis, five had no evidence of pulmonary fibrosis; and two had declining lung function. Two had mild allergies, one had high serum IgE, and all had normal serum tryptase levels. Healthy controls consisted of both males and females in their 2^nd^–4^th^ decades of life, with mild or no history for allergies and normal pulmonary function tests (PFTs). Written informed consent for protocol 04-HG-0211 was obtained from patients with HPS-1 pulmonary fibrosis or idiopathic pulmonary fibrosis undergoing lung transplantation to procure diseased lung tissue specimens.

**Table 1:** Demographics of HPS-1 patients.

Patient	Age	Pertinent medical history	HPS subtype	IgE (0–90 IU/ml)	Tryptase (< 11 ng/ml)
1	46	No fibrosis, allergies, normal PFTs	1	125.0	3.2
2	47	Moderate fibrosis, mild allergies, decreasing PFTs	1	69.8	7.0
3	36	No fibrosis, allergies, normal PFTs	1	1794.0	6.7
4	24	No fibrosis, allergies, normal PFTs	1	57.7	6.9
5	39	No fibrosis, allergies, decreasing PFTs	1	9.3	4.2
6	39	No fibrosis, mild allergies, normal PFTs	1	7.6	5.0

PFTs = Pulmonary Function Tests

### Culture of CD34+ derived human mast cells

Peripheral blood CD34^+^ progenitor cells were enriched to 90–95% by using commercially available affinity columns [9] and cultured in serum-free media (SFM) (StemPro-34; Life Technologies, Grand Island, NY) supplemented with 2 mM L-glutamine, 100 U/ml penicillin, 100 μg/ml streptomycin (complete SFM) and 30 ng/ml recombinant human (rh)IL-3 (first wk only), 100 ng/ml rhSCF and 100 ng/ml rhIL-6 [9]. Hemi-depletions, followed by replenishment with complete SFM and cytokines, were performed each wk. All cultures contained >95% HuMC by 6–8 wks. Kimura’s stain was used to visualize HuMC on a hemocytometer, and acidic toluidine blue, Wright-Giemsa, or anti-tryptase were used for cytopreparations (Cytospin 3; Shandon, Pittsburgh, PA).

### Tissue Histology

To examine dermal mast cell numbers and morphology, healthy subjects and HPS-1 patients underwent punch biopsies on the volar surface of the forearm. Skin was prepped, anesthetized with 1% lidocaine at the corners of a 2” x 2”area leaving the center uninfiltrated and 4 mm punch biopsies obtained and bisected. One bisection was fixed in 10% phosphate-buffered formalin and embedded in paraffin for sectioning and staining with anti-tryptase, H&E, Melan-A and Fontana-Masson. For immuno-histochemistry, 5 micron tissue sections were prepared and placed on poly-L-lysine coated slides. Immunohistochemistry was performed with a Ventana BenchMark ULTRA automated instrument (Roche Labs, Tucson, AZ). The primary antibody (anti-human mast cell tryptase, clone AA1, Dako, Catalog #M7052) was diluted 1:400 and incubated with tissue sections for 32 minutes followed by automated detection with a Ventana Ultraview immunohistochemistry detection kit (Roche Labs, Tucson, AZ). This detection kit utilizes a cocktail of enzyme-labeled secondary antibodies that detects mouse IgG and IgM and rabbit IgG primary antibodies. The chromogen used in the detection kit was alkaline phosphatase red. Histologic examination was performed with an Olympus BX41 microscope equipped with UPlanSApo objectives, and photomicrographs were captured with an Olympus DP71 digital camera using an Olympus DPController and DPManager softwares. The other bisection was fixed in 2.5% glutaraldehyde in 0.1 M sodium cacodylate buffer, ph7.3, for electron microscopy. Scoring of dermal mast cell number was performed under 400x magnification using an eyepiece graticule with a 10 x 10 matrix of 0.04 mm squares (Graticules LTD, Tonbridge, England) as described [10]. Wright-Giemsa staining of >99% normal control and HPS-1 HuMC cultures was performed using cytocentrifuged cell preparations as previously described [4].

To examine lung mast cell numbers and morphology, explanted lung tissue specimens (lung tissue specimens procured from lung transplantation) were fixed in 10% phosphate-buffered formalin, routinely processed, embedded in paraffin and stained with anti-tryptase.

### Electron Microscopy

Skin biopsy specimens were prepared for transmission electron microscopy as described previously [11]. More than fifty micrographs of each sample were taken and the ultrastructural granular patterns scored by 3 individuals blinded to the subjects; Twenty cells and at least 500 granules each from 5 normal controls and 5 HPS-1 patients were scored. Scanning electron microscopy (SEM) was performed to visualize matrix from control and transfected HPM clones. Briefly, 1 x 10^5^ HPM cells were suspended in 3 ml of complete SFM and seeded in 6 well plates; each well of which contained one silicon chip (Ted Pella, Inc, Rochester, NY). Hemi-depletions were performed twice weekly. Silicon chips were gently rinsed every two wks over 8 wks to remove adherent cells, washed twice in cold 1X PBS, fixed in 2.5% glutaraldehyde 0.1 M sodium cacodylate buffer, and post-fixed with 1.0% osmium tetroxide in 0.1 M sodium cacodylate buffer. Specimens were then dehydrated with a graded ethanol series, critical point dried under CO_2_ in a Bal-Tec model cpd 030 Drier (Balzers, Liechtenstein), mounted on aluminum studs, and sputter coated with 50 A of iridium in a model IBSe ion beam sputterer (South Bay Technologies, San Clemente, CA) prior to viewing at 5 kV in a Hitachi SU-8000 field emission scanning electron microscope (Hitachi, Tokyo, Japan).

### Electron Tomography

Two hundred nm sections of embedded skin biopsy specimens were collected on glow discharged carbon grids, and 10 nm colloidal gold fiducial markers were applied. Using a linear tilt scheme, single axis tilt series were collected on a Tecnai BioTwin Spirit TEM (FEI) operated at 120 kV. Images captured over a tilt range of +/- 68° (1° increments) at 1 μm defocus were recorded on a 2048 x 2048 pixel UltraScan 1000 Gatan CCD camera using the automated tomography acquisition software (Xplore 3D, FEI). Resulting images had a binning factor of 1 and pixel size of 2.1 nm. The tilt series were aligned using either Inspect 3D (FEI) or IMOD software package (version 4.2.5) and SIRT reconstructions of 35 iterations were performed. Movies were generated through the volume ortho-slices and volren models created from unfiltered tomograms with inverted contrast by thresholding colors based on pixel intensity within the volumes using the Amira Visualization Package (version 5.3.0, Visage Imaging, Carlsbad, CA).

### Derivation of HPS proMastocyte Cell Line

One typical HPS-1 HuMC culture at 5–6 weeks was eventually overgrown by a population of smaller, rapidly dividing cells. This culture was expanded and gradually weaned into flasks containing complete SFM without cytokines. Cells were dual-stained, selected for FcɛRI+/CD117+ expression, and single-cell cloned. Two clones, designated Hermansky-Pudlak (HP) proMastocyte (M) clones #3 and #4, had a myeloid or monocytic appearance and granularity and were expanded for further study.

### Ethics Statement

The description herein of the HPM cell line developed under protocols 98-I-0027 and 04-HG-0211 was permitted by the NIAID and NHGRI Institutional Review Boards in consultation with the Department of Bioethics. Written informed consent to publish and distribute the HPM cell line was obtained and is on file from the donor patient.

### Flow Cytometry

To determine HuMC expression of CD surface markers, normal and HPS-1 HuMCs were incubated in 1X PBS containing 0.1% BSA and 1% milk, followed by staining with conjugated mouse monoclonal antibodies to CD 63 (561925), 69 (555531), 117 (550412) or 203c (563297) (BD PharMingen, San Diego, CA) and human IgE (401152) (EMD Millipore, Billerica, MA) which had been biotinylated as described [12]. Cloned HPM cells were incubated in 1x PBS containing 0.1% BSA and 1% milk followed by staining with conjugated mouse monoclonal antibodies to CD1c (564900), 2 (563820), 3 (561803), 10 (555375), 11c (340713), 13 (340686), 14 (555398), 15 (555402), 16 (555407),19 (555412), 20 (555623), 22 (562859), 25 (555432), 33 (555450), 34 (555822), 36 (555455), 45 (555483), 49d (555503), 63 (561925), 64 (558592), 69 (555531), 117 (550412), 123 (561050), 203c (563297), CXCR4 (555975), BSP1 (552754) (BD PharMingen, San Diego, CA), EMR1 (sc-25829) (Santa Cruz Biotechnology, Dallas, TX) and biotinylated human IgE. Final dilution of antibodies ranged from 1:20–1:50. Cells were analyzed for intracellular proteases using conjugated monoclonal mouse antibodies to tryptase (MAB1222), chymase (MAB1254B) (Chemicon, Temecula, CA) and carboxypeptidase (gift of Andrew Walls, University of Southampton, UK) by flow cytometry and immunoassay [13]. For intracellular staining for tryptase, chymase and carboxypeptidase, cells were fixed with 4% paraformaldehyde and permeabilized [13]. Analyses were performed on at least 10,000 cells.

### Mediator Assays and β-Hexosaminidase (β-Hex) Release

Histamine content was determined using an enzyme immunoassay kit according to the manufacturer’s instructions (Cayman Chemicals, Ann Arbor, Michigan) and reported as pg/ml after normalizing for cell numbers. β-Hex and cytokine (GM-CSF, TNF-α, TGF- β, IL-6) release were assayed according to manufacturer’s instructions (R&D Systems, Minneapolis, MN) following crosslinking of FcɛRI as described [14]. In some experiments, HPM cultures were supplemented with 100 ng/ml rhSCF and rhIL-6 up to 4 wks to determine the effect on histamine content and β-Hex release. Control and HPS-1 HuMCs and HPM cell cultures were incubated overnight with 100 ng/ml biotinylated human myeloma IgE (bIgE) (BD PharMingen, San Diego, CA), followed by crosslinking with streptavidin (SA) (Sigma-Aldrich, St. Louis, MO). β-Hex release was reported as % of total cell content.

### Karyotype Analysis

To determine the karyotype of HPM cell cultures, metaphases were harvested from 5–10 x 10^6^ cells that were arrested in metaphase using colcemid overnight, placed in hypotonic potassium chloride, and fixed in methanol and acetic acid. Metaphases were stained with DAPI (Vectashield H-1200; Vector Laboratories, Burlingame, CA) and computer inverted to black and white images.

### Chemotaxis

Control, HPS-1 HuMCs and HPM cultured cells (1 x 10^5^ cells/sample) were incubated overnight in 2 mM L-glutamine, 100 U/ml penicillin, 100 μg/ml streptomycin (complete SFM) without cytokines. Cells were then washed twice and suspended in 100 μl complete SFM in the top wells of a 8 μm pore size Transwell plate (Costar, Tewksbury MA) for 30 min, with 600 μl complete SFM without cytokines or HEPES containing 0.5% BSA. The cells in the upper wells were then transferred to the test well containing 600 μl complete SFM supplemented with 10 ng/ml SCF or a negative control containing HEPES with 0.5% BSA. Wells were incubated 3–4 hrs. Cells and media in the lower chamber were then spun down, resuspended with 100 μl PBS and counted.

### Imatinib Induced Apoptosis

To determine if HPS-1 HuMCs would undergo apoptosis in the presence of imatinib [15], eight wk old control and HPS-1 HuMCs were cultured in the presence of 1 μM imatinib for 1 and 5 days, followed by flow cytometry for CD117, CD63, CD203c and annexin V. Analyses were performed on at least 10,000 cells.

### PCR of genomic DNA for *HPS1* 16-bp duplication (c.1470_1486dup16)

To determine if HPS-derived HuMCs expressed the *HPS1* 16-bp duplication, genomic DNA was isolated from 2 x 10^6^ cells using the QIAamp DNA Mini kit (QIAGen, Gaithersburg, MD) according to the manufacturer’s instructions. Targeted PCR was then performed with 100 ng DNA and the following primers: Forward primer 5’-GGTCCCTTCTGCTGTAATGC-3’, Reverse primer 5’-GCTGCGTGAAGGAAGTACG-3’. A thermal cycler was used at 94°C for 2 min to denature the template and activate the enzyme. PCR amplification was performed with 35 cycles as follows: 94°C for 30 sec, 55–65°C for 60 sec, 72°C for 30 sec, and 72°C for 7 min. Products were analyzed by gel electrophoresis using 8% acrylamide Novex TB gels (Life Technologies, Grand Island, NY) and visualized using ethidium bromide staining. The *HPS1* 16-bp duplication resulted in a band ~256 bp, in contrast with a normal control band of ~240 bp [16].

### Transfection and Transduction of HPM Cells

HPM clones #3 and #4 cultured in complete SFM alone or supplemented with rhSCF and rhIL-6 over 4 wks were transfected using Amaxa Nucleofector II (Lonza, Allendale, NJ). Briefly, 2 x 10^6^ HPM cells were washed and centrifuged at 90 x g for 8 min. HPM cells were resuspended in 100 μl of transfection medium (cell line Kit V) containing 2 μg of the appropriate cDNA plasmid construct and gently mixed. The mixture was placed into cuvettes and transfected using the aortic smooth muscle transfection program [17]. Cells were then plated out in pre-warmed complete medium and stored at 37°C in a humidified incubator for the indicated times. After 48 hrs, cells were maintained in a T25 flask containing complete SFM media supplemented with 100 ng/ml rhSCF, 100 ng/ml rhIL-6 and 1–4 μl/ml G418 Sulfate (50 mg/ml; Mediatech, Manassas, VA). Hemi-depletions with supplemented complete media were performed wkly for expanding cultures. Clones transfected without prior treatment with rhSCF and rhIL-6 did not survive transfection and G418 selection. Lentiviral approaches were employed to achieve effective overexpression and transduction into a permanent cell line. Preliminary transfection experiments using HPM clone #4 and prior treatment with rhSCF and rhIL-6 in addition to Amaxa Nucleofector II gave optimal efficiency expressing *HPS1*. Thus, *HPS1* overexpression in HPM clone #4 was performed using GeneCopoeia third generation HIV-based lentiviral vector system and human HPS-1 ORF cDNA lentiviral particles, according to the manufacturer’s instructions. HPM clone #4 was examined pre- and post-transduction for HPS-1 protein, granulation, degranulation, migration, survival and proliferation.

### Extracellular Matrix

To analyze matrix components, nonadherent HPM control and transfected cells in T75 flasks were first counted and removed by pipetting. Matrix adherent to flask surfaces was washed twice for 5 min with cold hypotonic (1:10) PBS with protease inhibitors to lyse adherent cells. Two hundred μl of PBS was then added and matrix removed with a Costar cell scraper (#3010; Corning Inc, Corning, NY). The matrix suspension was centrifuged at 2400 RPM x 5 min, decanted and 100 μl of 1x PBS and 100 μl of 2x sample buffer (SB) were added. Matrix suspension was kept at -80°C until ready for Western blotting. Lysates from non-adherent cells for Western blotting were prepared from 1 x 10^6^ cells as described [18]. Twenty μl of each cell lysate which is equal to 1.25 x 10^5^ cells and 25 ul of matrix suspension was then loaded on to 4–12% polyacrylamide gels. Proteins were separated by electrophoresis and transferred to nitrocellulose membranes. Membranes were blocked for 60 min using Odyssey blocking buffer (LICOR Biosciences, Lincoln, NE) and incubated overnight with the primary antibodies. After 60 min incubation with the IR-dye (680RD or 800CW)-labeled secondary antibodies (1:20,000), immunoreactive proteins were visualized using an Odyssey imager (LI-COR Biosciences).

### Immunofluorescence and Confocal Microscopy

Control and transduced HPM cells were cultured for 6 wks on coverslips placed in 6 well plates, at which time coverslips were removed and gently pipetted to remove adherent cells. Coverslips were washed twice in cold 1x PBS and fixed for 45 min at 4°C in 4% PFA. Coverslips were washed three times in cold PBS and blocked overnight at 4°C in PBS containing 5% BSA (fraction V). Coverslips were then stained with rabbit polyclonal anti-fibronectin or isotype control in PBS containing 1% BSA for 45 min in a humidified incubator, washed four times with cold 1x PBS, and AF594 conjugated goat anti-rabbit antibody applied for 30 min in a humidified incubator. Next, coverslips were washed four times in cold 1x PBS and mounted in ProLong Gold anti-fade reagent (Molecular Probes, Life Technologies, Grand Island, NY). Confocal microscopy was performed on a Leica TCS SP8 confocal microscope. Images were collected using a 63x oil immersion objective NA 1.4, and acquired using a HyD detector and without digital zoom. A stack of images (200 nm thickness) was taken for each high power field from the interface of the coverslip through the depth of staining. Snapshots were taken using Imaris x64 v7.6.3 of the composite Z projections.

### Gene Microarrays and Analysis

Analysis of differential gene expression from 8 wk old control and HPS-1 HuMCs and HPM cells was performed using cDNA generated from cultured cells. Isolated RNA (4 μl at >50 ng/μl concentration) was labeled with the 3’ IVT Express labeling kit. Sample QC was hybridized and analyzed on an Agilent Bioanalyzer using an Affymetrix GeneChip Human Genome U133 Plus 2.0 array. The patient sample size was run in triplicate (n = 3). Microarray analysis was performed with the use of Bioconductor R statistical language packages. For statistical computations, R version 3.1.0 (2014-04-10) was used, and Bioconductor version 2.14 was used as an open source suite for tools that perform background adjustment, normalization and summarization, and differential gene expression. For post-filtering analysis, custom scripts were used. Quality control was done with the Affy 1.42.3 package. The Variance Stabilization and Normalization (VSN) 3.32.0 package was used to compare scatter plots of raw and normalized data to assess the quality of the data. The shape of the data distributions of all samples was compared as well as the relationships of the mean and variances. Two different methods, Robust Multi-array Average (RMA) and VSN, were used for normalization. When there was at least 80% overlap between the two methods, the VSN list was used. The overlap in lists was determined by comparing differentially expressed genes in top ranking criteria using the Linear Models for Microarray (limma) 3.20.9 package output (adjusted P-value < 0.05 and FDR < 0.05). Differentially expressed gene lists were initially separately calculated for VSN and RMA. These lists were then compared with each other as follows: for background adjustment, normalization and summarization, the RMA method was applied separately and used as input for limma. VSN was used separately to produce normalized data where the variance was dependent on the mean intensity, and where a statistical transformation was applied based on a stabilization of the variance. VSN output was also separately processed by limma to identify differentially expressed genes by the use of linear models. When both methods agreed with at least 80%, the differentially expressed gene list based on VSN normalization was used. The VSN—limma output with differentially expressed genes was filtered based on statistical significance with FDR values lower than 0.05. The FDR-filtered list was used as input into pathway and network analysis with IPA Ingenuity Pathway Analysis (www.ingenuity.com). In IPA, the functional analysis workflow was used to identify the enriched fibrosis functional groups, filtered based on p-value and activation z-score. The option to analyze Causal Networks was enabled upon running the core analysis. From the analysis options, 'functions and diseases', 'upstream analyses’, 'upstream regulators' and 'causal networks' were exported to excel spreadsheets for post-filtering analysis.

### Statistical Analysis

Statistical significance of differences was performed using the two-tailed unpaired Student’s t-test. Differences were considered to be significant when p<0.05. Data was expressed as means ± SEM.

## Results

### Mast Cells Localize to the Fibrotic Alveolar Interstitium in HPS-1 Lungs

**Fig 1:**
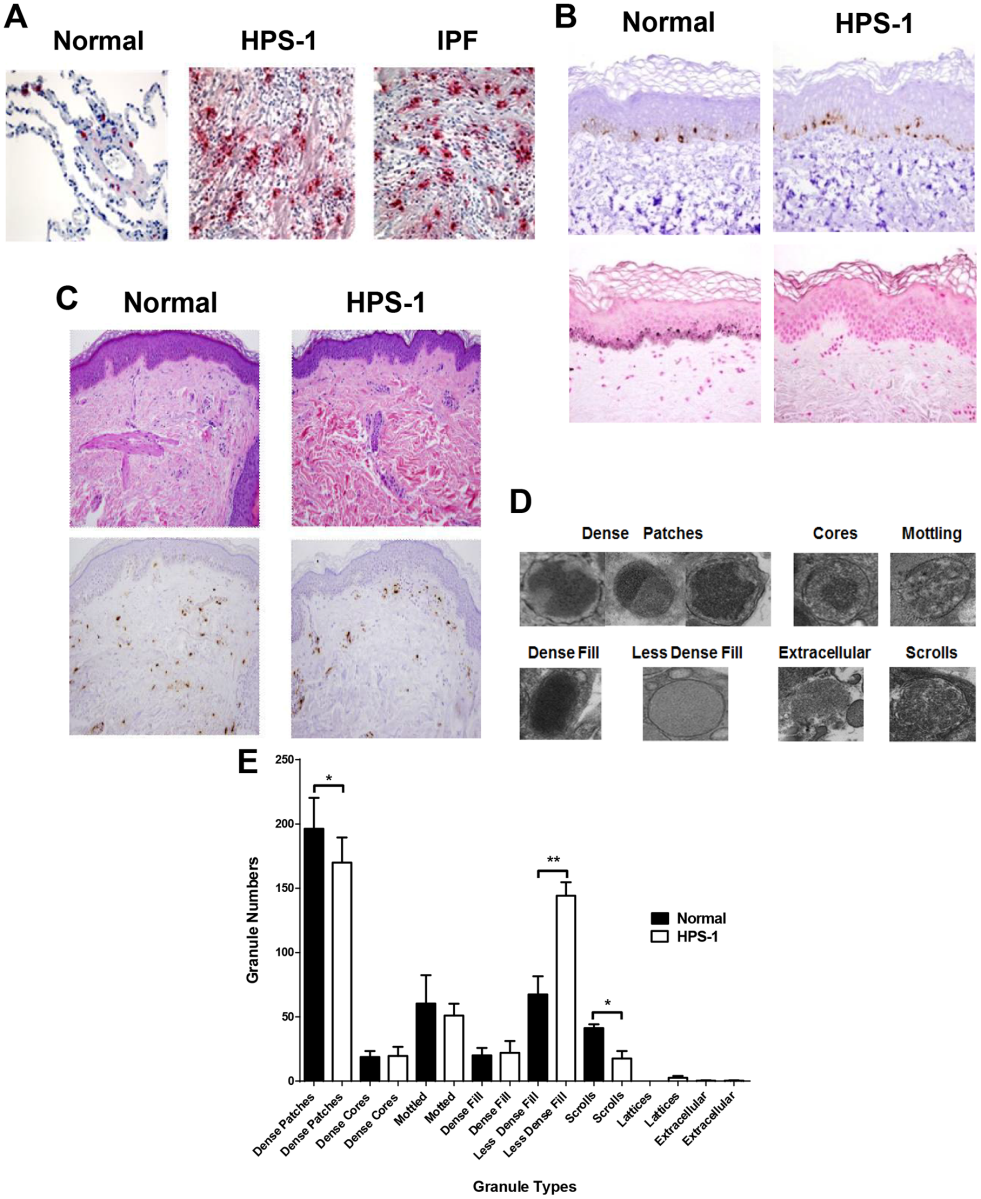
Pulmonary mast cells localize to fibrotic regions of the lung parenchyma in HPS-1 pulmonary fibrosis. Histology, immunohistochemistry and ultrastructure of HPS-1 derived dermal mast cells. A) Representative anti-tryptase stained lung sections from patients with HPS-1 pulmonary fibrosis and IPF showed significant numbers of pulmonary mast cells within fibrotic alveolar interstitium. Mast cells are shown in normal lung tissue for comparison. Data are representative of four separate patients with either HPS-1 or IPF; B) Staining of skin biopsies reveals normal numbers of melanocytes (Melan-A) (upper panels) but decreased melanin pigmentation (Fontana-Masson) in HPS-1 patients (lower right panel; compare with lower left panel); (C) Wright-Giemsa staining (upper panels) and tryptase staining (lower panels) of dermal mast cells; (D) Ultrastructure of normal and HPS-1 HuMC granules used for classification include dense patches, cores, mottling, dense fill, less dense fill and extracellular patterns, with or without scrolls or lattices, and E) Comparison of normal and HPS-1 granules showed dense patches with scrolls were significantly increased in normal mast cell granules as compared with less dense fill increased in HPS-1 granules, when granules were scored by three independent observers blinded to the identity of originating mast cells. Data are the means ± SEM (n = 3). *p < 0.05, **p < 0.01.

We first determined the general distribution of HuMCs in the explanted lungs of HPS-1 patients. Lung tissue sections from patients with advanced HPS-1 pulmonary fibrosis and IPF show numerous tryptase positive HuMCs (Fig 1A). HuMCs were most prominent in the alveolar interstitium within fibrotic regions of the lung parenchyma from patients with HPS-1 pulmonary fibrosis and IPF. HuMC localization, density and morphology appear similar in HPS-1 pulmonary fibrosis and IPF. Higher magnification of HPS-1 lung as seen in Fig 1A (S1 Fig) reveals red, tryptase positive extracellular granules (small arrows) and tryptase positive HuMCs (large arrows) with a circumferential reddish “blush” of extracellular tryptase, together suggesting that these HuMCs are activated and releasing tryptase. In normal lung tissue, HuMCs are fewer in number and localize to the alveolar walls and peri-bronchiolar regions.

### HPS-1 Dermal Mast Cells Express the HPS Phenotype

HPS-1 and normal skin biopsies are similar in a variety of parameters. They both exhibit basal layer melanocytes (Fig 1B, upper panels) and tryptase-positive dermal mast cells of similar gross morphology and number (Fig 1C; S2 Fig). However, the HPS-1 skin biopsies show decreased melanin pigmentation within basal layer epidermal keratinocytes on light microscopy (Fig 1B, lower panels) and distinct ultrastructural variations in mast cell granules by electron microscopy (Fig 1D and 1E, S3 Fig, S1 and S2 Videos) [19]. Specifically, HPS-1 dermal mast cells exhibit increased numbers of granules displaying the less-dense fill pattern (p<0.01), decreased numbers of dense patches (p<0.05) and reduced scroll figures (p<0.05). Hence, HPS-1 dermal mast cells express a granule defect detectable in skin biopsies.

### Mast Cells Cultured from HPS-1 Patients Exhibit Constitutive Activation and Impaired Secretory Responses

**Fig 2:**
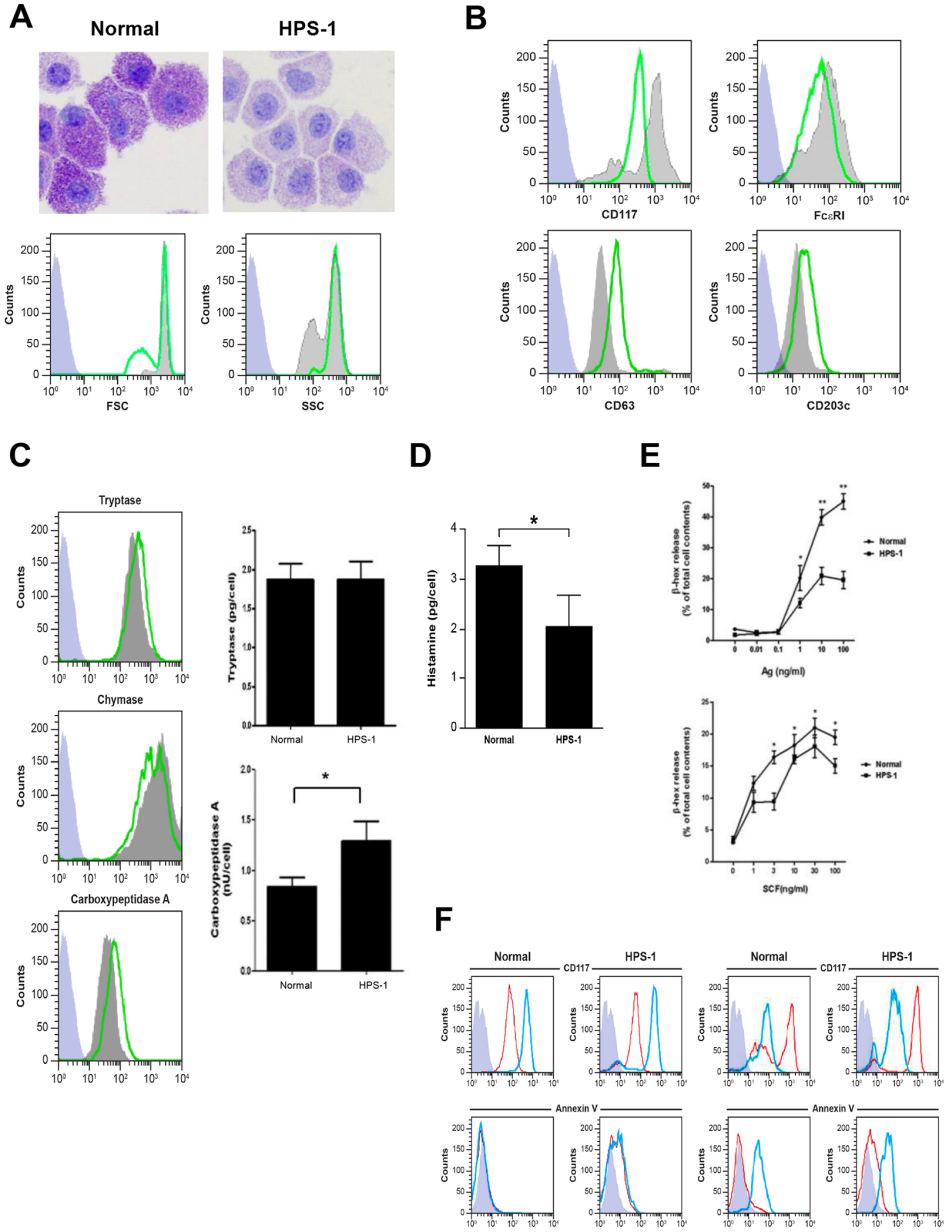
Characteristics of normal and HPS-1 HuMCs. A) Wright-Giemsa staining of normal (left panel) and HPS-1 (right panel) HuMCs. HPS-1 granules exhibit less dense staining. Representative flow cytometry of size (FSC) and granularity (SSC) of HuMCs. Grey-shaded histograms are normal HuMCs, green lines represent HPS-1 HuMCs, blue-shaded histograms are isotype controls; B) Representative flow cytometry of CD surface marker histograms of HuMCs. Grey-shaded histograms are normal HuMCs, green lines represent HPS-1 HuMCs, blue-shaded histograms are isotype controls. CD117 and FcɛRI expression of the CD117^high^/FcɛRI+ population is reduced in HPS-1 HuMCs. CD63 and CD203c activation marker expression is increased; C) Representative flow cytometry of permeabilized HPS-1 HuMCs (histograms) shows tryptase and carboxypeptidase staining is somewhat increased in HPS-1 HuMCs but not chymase staining. Grey-shaded histograms are normal HuMCs, green lines represent HPS-1 HuMCs, blue-shaded histograms are isotype controls. HPS-1 HuMC carboxypeptidase but not tryptase content (bar graphs) is increased based on protein assays; data are the means ± SEM (n = 3). *p<0.05. Histograms (A-C) are representative of at least 3 separate experiments; D) Histamine content (pg/cell) of HPS-1 HuMCs is reduced when compared with normal HuMCs; E) β-Hex release (upper graph) of HPS-1 HuMCs was reduced at higher concentrations (>1 ng/ml) of antigen when compared with normal HuMCs. β-Hex release (lower graph) was enhanced but at reduced levels for HPS-1 HuMCs in the presence of 0.1 ng/ml antigen with increasing concentrations of SCF. Data are the means ± SEM (n = 3) (D-E). *p<0.05, **p<0.01; F) Representative flow cytometry of CD117 and annexin V expression of normal and HPS-1 HuMCs incubated with imatinib for 24 hrs (left-sided histograms) and 5 days (right-sided histograms). Red lines are untreated HuMCs, blue lines are imatinib-treated cells, blue-shaded histograms are isotype controls. Histograms are representative of 2 separate experiments performed in duplicate.

Primary CD34+ derived HuMCs from HPS-1 patients maintain expression of the *HPS1* mutation in culture (data not shown). After 8 weeks in culture, HPS-1 HuMCs granules stain less intensely compared to normal HuMCs (Fig 2A, upper figures), though size and granularity were similar to normal HuMCs (Fig 2A, histograms). In addition, CD117 expression is markedly reduced and FcɛRI expression somewhat reduced in HPS-1 HuMCs compared to control HuMCs (Fig 2B, upper histograms), while expression of the surface activation markers CD63 and CD203c is increased in HPS-1 HuMCs compared to normal cells (Fig 2B, lower histograms). Expression of both intracellular carboxypeptidase and tryptase is somewhat increased in HPS-1 HuMCs compared to normal HuMCs (Fig 2C, histograms); while chymase expression is generally similar in HPS-1 and control cells. Assays of protease content show higher levels of cellular carboxypeptidase, but not tryptase, in HPS-1 HuMCs compared to control cells (Fig 2C, bar graphs). The histamine content of HPS-1 HuMCs is approximately half that of normal control HuMCs (Fig 2D). GM-CSF, TNF- α, TGF- β, and PGD_2_ production with and without FcɛRI crosslinking is similar between normal and HPS-1 HuMCs (S4 Fig). β-Hex release from HPS-1 HuMCs compared to normal HuMCs is significantly reduced at higher concentrations of antigen, although HPS-1 HuMC β-Hex release increases in a dose-dependent manner when incubated with SCF (Fig 2E). Furthermore, reduced amounts of β-Hex release in the presence of low concentration of Ag and increasing amounts of SCF are consistent with an internal difference in exocytic capacity distinct from FcɛRI aggregation.

### Effect of Imatinib on HPS-1 HuMCs

To determine whether HPS-1 HuMCs would undergo apoptosis in the presence of imatinib, HuMCs were incubated for 1 and 5 days with 1 μM imatinib and CD surface marker expression and apoptosis were analyzed. As seen in Fig 2F, following 24 hrs incubation (left-sided panels), enhanced expression of CD117 is noted in both control and HPS-1 HuMCs without increased surface expression of annexin V. However, following 5 days incubation (Fig 2F, right-sided panels), CD117 expression is reduced concurrent with increased expression of annexin V. Thus, HPS-1 HuMCs undergo apoptosis in the presence of imatinib, suggesting a possible role for this agent in vivo.

### Generation and Phenotype of a Hermansky-Pudlak proMastocyte (HPM) Cell Line

In one HPS-1 HuMC culture, immature appearing cells overgrew more typical, mature appearing cells and became the only surviving cell population. Based on flow cytometry staining (see below), these cells closely resembled mast cell progenitors, i.e. promastocytes. These Hermansky-Pudlak proMastocyte (HPM) cells were expanded and weaned from dependence on rhSCF and rhIL-6. HPM cells were dual-stained, selected for FcɛRI+/CD117+ expression, and single-cell cloned. Two clones #3 and #4 had a myeloid or monocytic appearance, granularity and expressed the *HPS1* mutation. Cells doubled every 3–4 days, displayed a normal 46XY karyotype (data not shown), and were generally spherical or oval-shaped with pleomorphic nuclei and occasional nucleoli. HPM cells measured 8–10 μm in diameter with rough surfaces and cytoplasmic projections, sparse cytoplasmic granules with vesicular bodies, electron dense material, and partial scroll patterns, consistent with an immature mast cell phenotype (S5 Fig).

**Fig 3:**
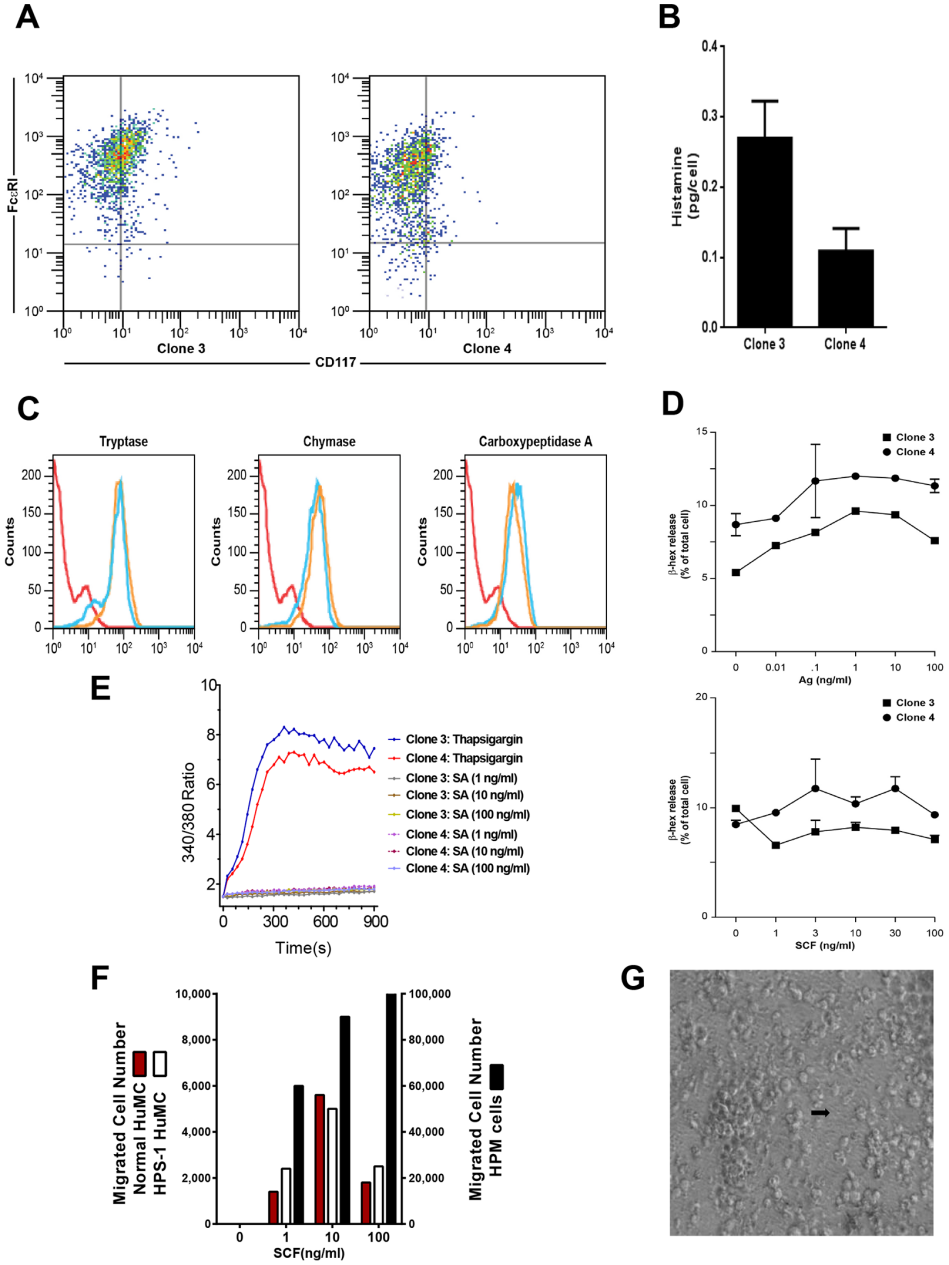
Characteristics of HPM cells. A) Representative dot plots of HPM clones show that HPM cells are FcɛRI+/CD117^low/-^; B) Histamine content of HPM clones ranges from 0.1–0.3 pg/cell; C) Representative flow cytometry from clone #4 of permeabilized HPM cells in the presence (blue line) or absence (yellow line) of rhSCF and rhIL-6 shows similar expression of tryptase, chymase and carboxypeptidase; D) β-Hex release (upper graph) of HPM clones #3 and #4 at 1 ng/ml antigen is minimum and ranged from 3–5% following crosslinking with antigen. In the presence of 0.1 ng/ml Ag, the addition of increasing concentrations of SCF does not enhance release (lower graph) from either clone. Data are from 2 separate experiments performed in duplicate (A-D); E) Thapsigargin but not FcɛɛRI crosslinking with SA induces calcium influx in HPM clones; F) In contrast to low migration of normal (red column) and HPS-1 (white column) HuMCs to all concentrations of SCF, representative chemotaxis of HPM clone #4 shows HPM migrated cell numbers (black column) are significantly elevated and show increasing migration to increasing concentrations of SCF. Data are representative of 2 separate experiments performed in duplicate (E-F), and G) Representative light microscopy of extracellular matrix formation in HPM clones 3 and 4 (arrow–matrix).

Several lines of evidence indicate that HPM clones #3 and #4 represent a credible model of early HPS-1 HuMCs. FACS analysis shows a single uniform population with strong positivity for FcɛRI and low staining for CD117 (Fig 3A). The HPM clones express HLA-DR, CD13, CD36, CD49d, and CD63, and to a lesser degree CD3, CD25, CD34, CD69, CD117 and CXCR4 (data not shown). Basophil BSP-1, myeloid dendritic markers CD1c and CD11c, and CD2 are negative (data not shown). Histamine content ranges from 0.1–0.3 pg/cell (i.e., 10–20 fold lower than in cultured mature HuMCs) (Fig 3B). Both HPM clones are greater than 95% positive for tryptase, chymase and carboxypeptidase, and protease content is unaffected by growth in rhSCF and rhIL-6 as shown by representative data from clone #4 (Fig 3C). Tryptase and chymase, but not carboxypeptidase, content are reduced when compared with HPS-1 HuMCs (compare Figs 3C and 2C, S6 Fig). In HPS, the melanosomes of melanocytes, dense granules of platelets, and lamellar bodies of type 2 pneumocytes have been studied fairly extensively, but little is known about HuMC granules, which are also lysosome-related organelles. Thus, HPM clones #3 and #4 express myeloid and activation markers and proteases consistent with a mast cell lineage, and they do not express basophil or myeloid dendritic cell markers. β-Hex release from the HPM clones is minimum (3–5%) in the presence of antigen, and the addition of SCF to antigen does not enhance release (Fig 3D). Consistent with low β-Hex release seen with antigen-induced FcɛRI crosslinking or PMA + ionomycin (S7 Fig), calcium influx is induced by thapsigargin, but not FcɛRI crosslinking (Fig 3E). Despite low surface expression of CD117, HPM cells strongly chemotax to SCF compared to mature control or HPS-1 HuMCs (Fig 3F). In total, the HPM cells resemble early mast cells with low levels of histamine and β-Hex and ability to undergo chemotaxis.

Cytokine release from HPM cells was compared with release from LAD2 cells, a human mast cell line closely resembling primary HuMCs. HPM cells constitutively release higher concentrations of IL-6, IL-8, GM-CSF, TNF-α, but not TGF-β (Table 2, second and last columns). Consistent with these data, concentrations of IL-6 in serum are significantly higher in patients with HPS-1 compared to normal volunteers (S8 Fig), and the HPS-1 patient with the highest level of IL-6 is the source of HPM clones. These data suggest that HuMC-derived IL-6 may contribute to elevated serum IL-6 levels in these HPS-1 patients. In other experiments, it was noted that flask surfaces of HPM clones become coated with extracellular matrix to which cells in suspension attached and proliferated (Fig 3G).

**Table 2:** Cytokine production by mock and *HPS1* transduced HPM cells.

Cytokine	Mock Transfected	HPS1 Transfected	LAD2 cells
IL-6 (pg/ml)			
3 days	61.54 ± 1.74	15.53 ± 0.01	17.12 ± 2.54
7 days	219.43 ± 6.15	24.43 ± 0.96	19.21 ± 0.23
IL-8 (pg/ml)			
3 days	89.26 ± 4.24	30.51 ± 1.28	21.68 ± 0.08
7 days	476.64 ± 13.94	125.42 ± 1.73	31.77 ± 0.00
GM-CSF (pg/ml)			
3 days	0	0	0
7 days	24.85 ± 1.61	28.92 ± 1.81	0
TNF-α (pg/ml)			
3 days	44.28 ± 0.27	54.59 ± 15.32	8.38 ± 3.34
7 days	194.94 ± 23.84	155.20 ± 9.97	24.46 ± 1.24
TGF-β (pg/ml)			
3 days	7.45 ± 0.26	7.61 ± 0.04	17.98 ± 6.00
7 days	9.24 ± 0.00	8.44 ± 0.41	18.46 ± 0.33

### Rescue of the HPS-1 Phenotype of HPM Clones

**Fig 4:**
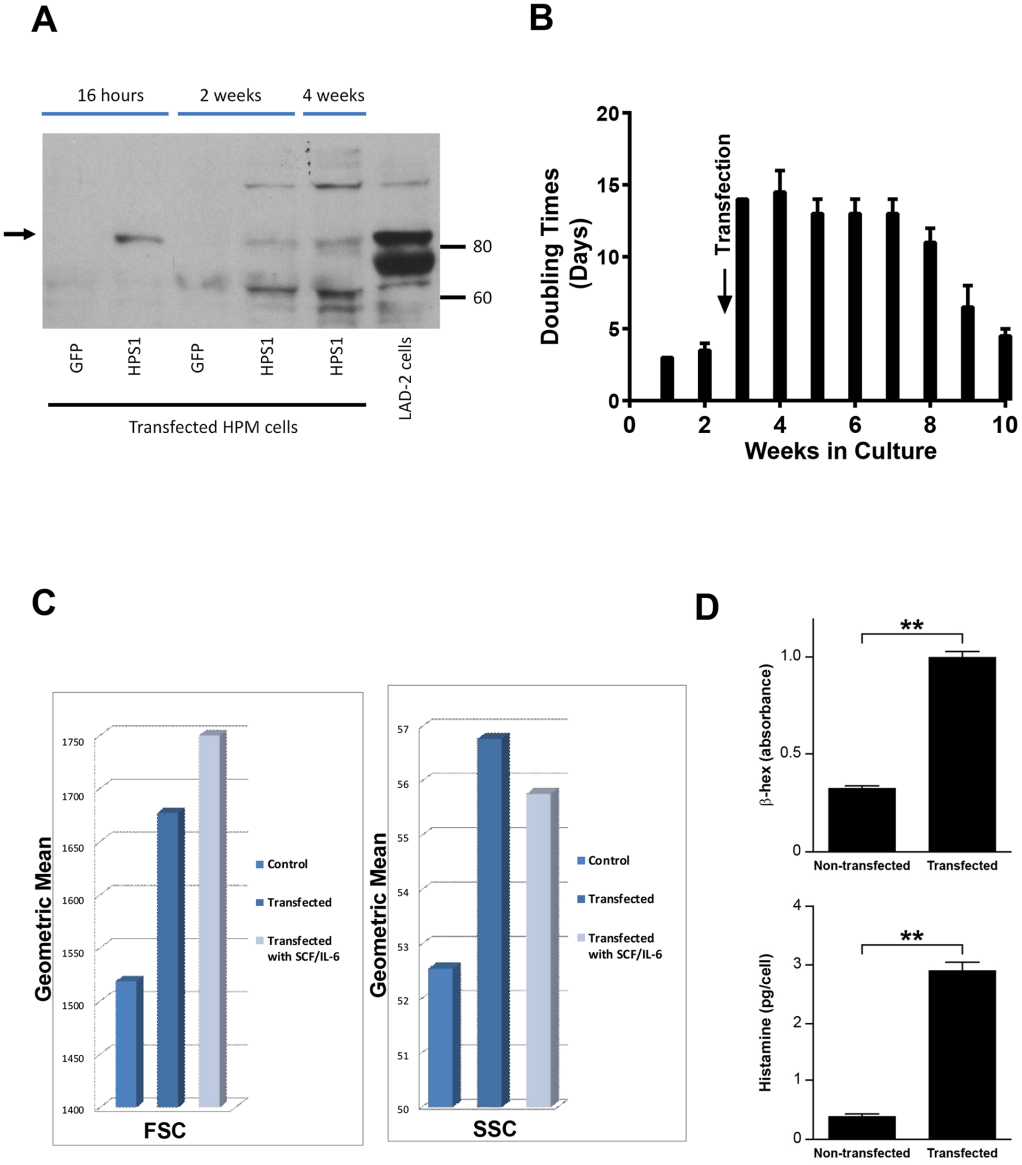
Results of transfection of HPM cells with normal *HPS1*. A) Representative Western blot of *HPS1* transfected HPM cells and control LAD2 cells demonstrating HPS-1 protein expression (arrow) in 16 hr, 2 and 4 wk HPM cultures, confirming transfection and expression of HPS-1 protein. Immunoblot is representative of at least 2 independent experiments; B) Slowing of doubling time 5–7 fold immediately following transfection with *HPS1*. Transfected cells reached average doubling rate of 3–4 days by 10 wks; C) FSC and SSC changes in HPM cells 4 wks following transfection. No additional changes were noted following supplementation of cultures for 4 wks with rhSCF and rhIL-6. Data are representative of 2 separate experiments performed in duplicate; D) Three-fold increase in cellular β-Hex content following transfection (upper graph) and ten-fold increase in cellular histamine content 4 wks following transfection (lower graph). Data are the means ± SEM (n = 3). **p<0.01.

To demonstrate the elements of the HPM phenotype related to the *HPS1* mutation, we first transfected HPM clones #3 and #4 with wild type *HPS1*. HPM clone #4, pretreated in culture with rhSCF and rhIL-6, gave optimal transient transfection efficiency and expressed HPS1 protein as early as 16 hours following transfection (Fig 4A). Western blot showed HPS1 protein expression at 2 and 4 weeks (Fig 4A). Transfection with *HPS1* slowed cell doubling times from 2–3 days to 14 days over approximately 5–6 weeks (Fig 4B). The cells became larger and more granulated, and flow cytometry demonstrated increases in FSC and SSC following transfection with and without incubation with rhSCF and rhIL-6 (Fig 4C). β-Hex and histamine cell contents were increased significantly by 4 weeks following transfection (Fig 4D). Stable transduction of HPM clone #4 was achieved using a lentiviral vector system with human *HPS1* cDNA. β-Hex release remained low, i.e., 5–15% (data not shown). Transduction of *HPS1* reduced and nearly normalized secretion of IL-6 and IL-8, but not GM-CSF or TNF-α (Table 2, column 3).

**Fig 5:**
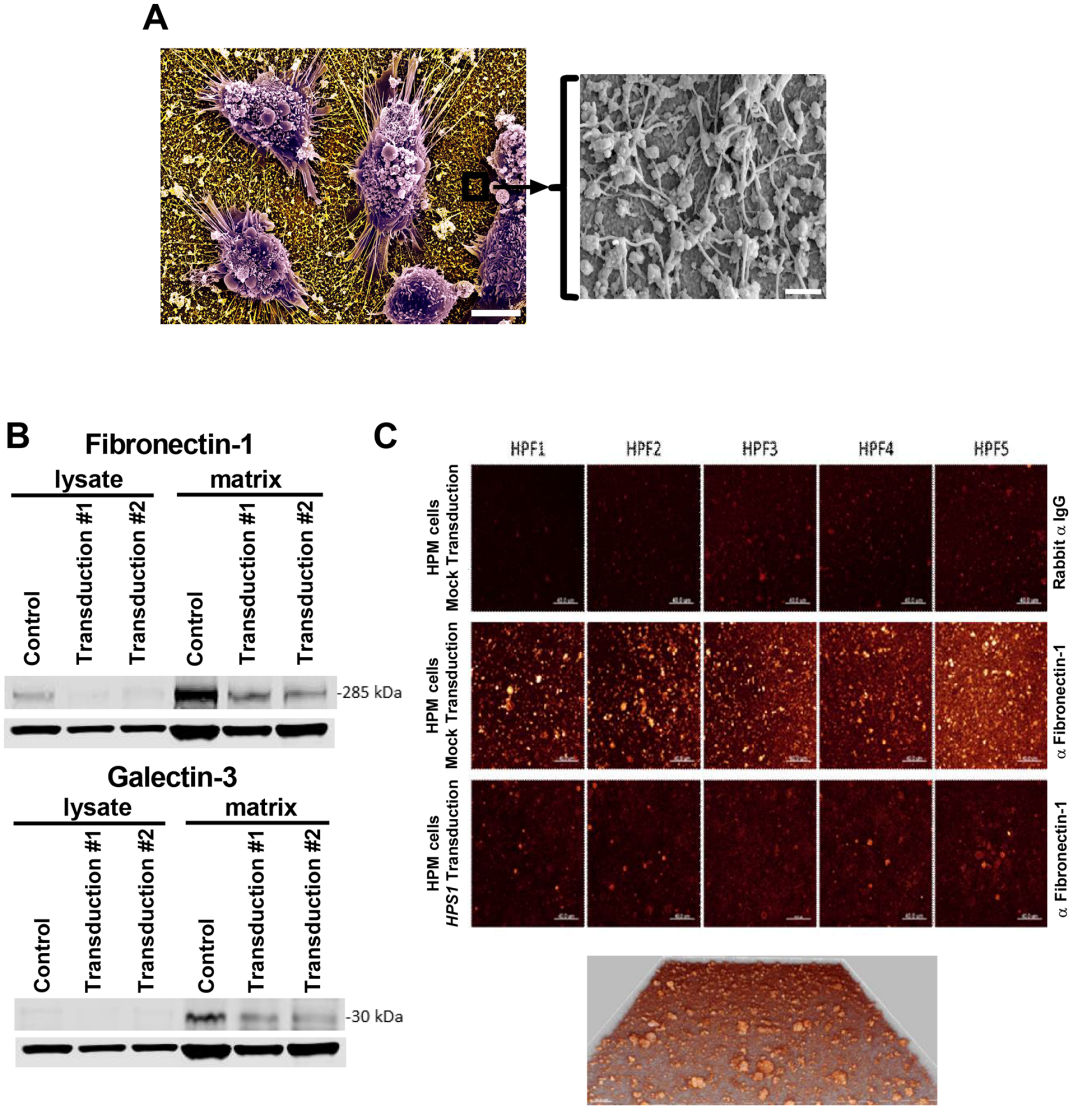
Analysis of extracellular matrix production. A) Scanning electron micrograph of HPM cells adherent to silicon glass surfaces and producing matrix; scale bar = 5 microns. Window shows higher magnification of surface-bound globular and fibrillar matrix structures; scale bar = 0.5 microns; B) Western blots of non-adherent cell lysates and matrix from mock transduced (control) and two independent transductions of HPM clone #4 with wild type *HPS1*. Proteins in the matrix reduce following *HPS1* transduction when compared with control, but no difference is seen between the two transductions. Lower rows in both blots are β-actin control for cell lysates. A single blot is presented that was probed with antibodies directed against fibronectin-1, galectin-3 and actin. Data are representative of 3 separate experiments; C) Confocal microscopy showing production of fibronectin-1 by mock transduced HPM cells labeled with anti-fibronectin-1 (middle panels) but not rabbit isotype control (upper panels). Significant reduction is noted following transduction with *HPS1* (lower panels). Five representative high power fields (HPF) are shown. All HPFs were acquired with identical parameters so that a direct comparison of intensity can be drawn. LUT was adjusted to see the intensity clearer and increased the brightness on all the panels equally. A 3D image of matrix from middle panels in (C) is shown at magnification 630X and shows depth of the matrix.

HPM clone #4 transduced with *HPS1* produced less matrix on flask surfaces than cells transduced with a mock vector (data not shown). Scanning electron microscopy of mock-transduced HPM cells grown on silicon chips showed adherent cells with surface-bound globular and fibrillar structures (Fig 5A). Western blot analysis of cell lysates and culture-bound matrix from mock-transduced HPM cells confirmed that production of fibronectin-1(FN-1) galectin-3 (LGAL3) was detected in the matrix but not in the cell lysate. For both FN-1 and LGAL3, production was reduced following transduction with *HPS1* (Fig 5B). HPM cells are strongly adherent and attach via filopodia to the matrix they secrete (Fig 5A); thus, the detection of actin in the Western blot analysis is likely due to the persistence of filopodia with their internal actin filaments following cell lysis [20–22]. Consistent with these data, confocal microscopy of matrix showed FN-1 immunostaining in mock transduced HPM cells; the levels were reduced following transduction with *HPS1* (Fig 5C).

### Microarray Gene Chip Analysis and Functional Groups

**Fig 6:**
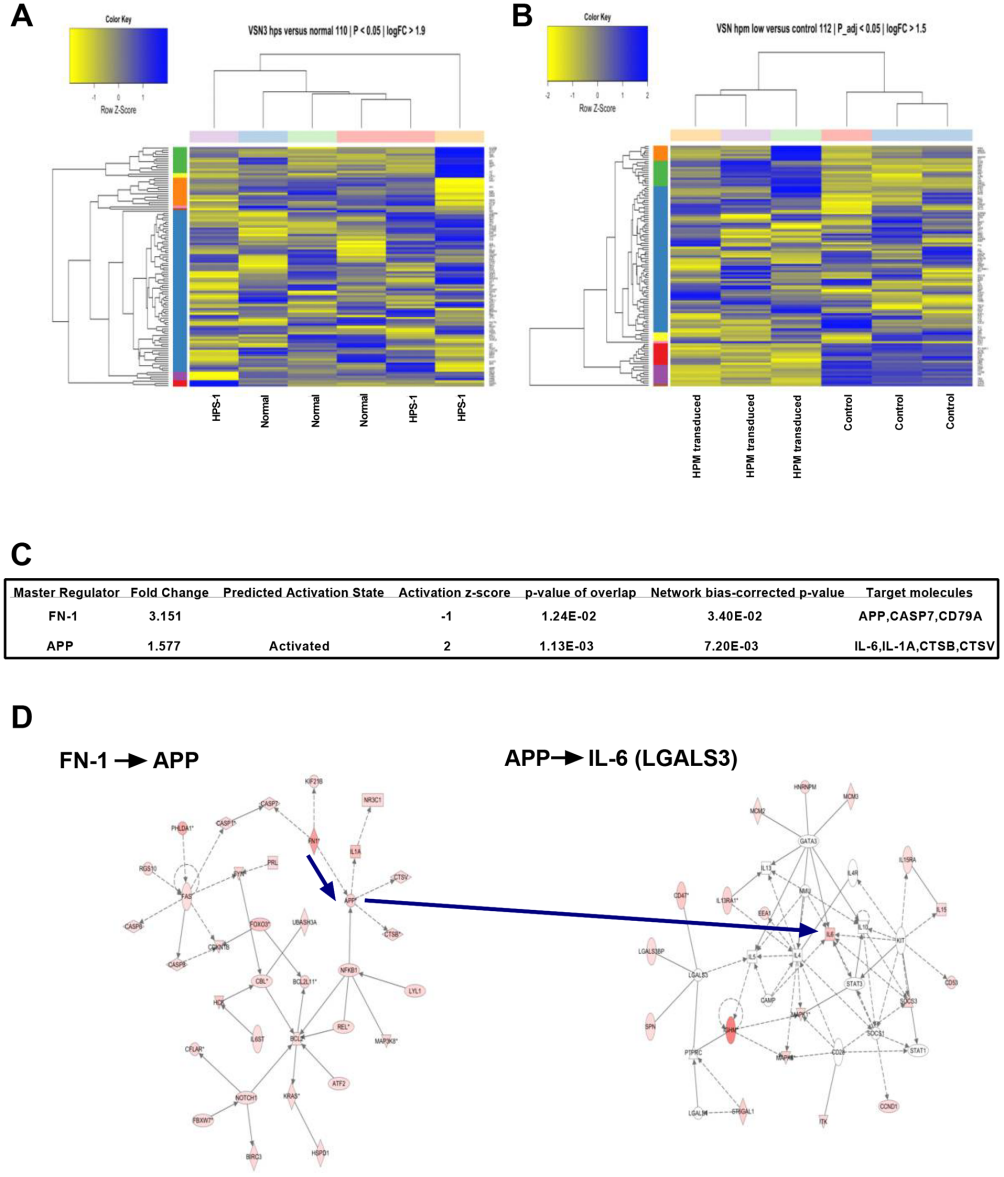
Differential gene expression and statistically significant networks. A) Heat map display of the strongest, differentially expressed genes measured in HPS-1 HuMCs compared with normal HuMCs; B) Heat map display of mock transduced (control) versus *HPS1* transduced HPM cells showing significant differentially expressed and enriched genes; C) FN-1 is a master regulator of amyloid precursor protein APP, CASP7, CD79A and ZBTB7A. Target molecules in the dataset found for APP were CTSB, CTSV, IL-1A and IL-6; D) Central to the FN-1 and APP relationship (left-sided diagram) is a network that in its turn targets the IL-6 network (right-sided diagram), all of which are significantly upregulated. Genes are represented in the nodes and gene-gene relationships in the edges. The intensity/color is a measure of the extent of differential expression. The shapes of nodes represent categories of gene function; FN-1 is an enzyme and shown as a diamond, IL-6 is a cytokine and shown as a square. IL-6 operates in the context of KIT, STAT3, SOCS1, GATA3 and CAMP.

To further understand the role of the HPS-1 defect in HuMC development and matrix production, we used microarrays for large-scale analysis of RNA isolated from purified normal and HPS-1 HuMCs and HPM mast cells. The Variance Stabilization and Normalization (VSN) 3.32.0 package and Robust Multi-array Average (RMA) methods across replicates were used for normalization of the arrays, and Linear Models for Microarray (limma) to generate differentially expressed gene lists based on triplicate statistics. Heat maps of genes differentially expressed were compared for normal and HPS-1 HuMCs (Fig 6A, Fig A in S9 Fig), and genes enriched and differentially expressed when mock transduced HPM cells were compared with *HPS1* transduced HPM cells (Fig 6B, Fig B in S9 Fig). Comparison of both heat maps showed a significant enrichment in the functional groups reflecting fibrogenesis, differentiation of connective tissue, fibrosis of tissue, growth of connective tissue and degranulation of cells (Table 3). Microarray analysis also indicated up-regulation of collagen-5A2, laminin-3, FN-1 and LGALS3 in HPM cells. Thus, genes involved in fibrogenesis are upregulated in HPS-1 HuMCs and mock transduced HPM cells, and transduction with *HPS1* cDNA decreased expression of genes associated with production of matrix components and cytokines. Employing a causal network analysis, we found modules revolving around FN-1 and amyloid beta (A4) precursor protein (APP) that appeared crucial for understanding their "target-genes" (Fig 6C). Specifically, the axes FN-1-> APP and APP -> IL-6 were statistically significant (Fig 6D).

**Table 3:** Functional groups enriched significantly in cultured HPS-1 HuMCs and non-transduced HPM cells.*

Functional Annotations	#Molecules	p-Value	Predicted Activation State	Activation z-score
Interphase of fibroblasts	26	1.56E-07	Increased	3.554
Morphology of cells	461	5.81E-13	Decreased	-2.789
Abnormal morphology of cells	311	1.92E-10	Decreased	-3.087
Activation of cells	237	1.03E-09	Increased	6.783
Fibrogenesis	98	2.99E-06	Increased	4.788
Degranulation of cells	59	2.34E-06	Increased	2.290
Proliferation of fibroblast cell lines	119	5.24E-11	Increased	3.129
Proliferation of fibroblasts	94	6.47E-09	Increased	2.157
Differentiation of connective tissue	175	7.92E-12	Increased	2.409
Differentiation of connective tissue cells	155	2.54E-11	Increased	2.500
Formation of connective tissue cells	46	2.15E-07	Increased	2.692
Proliferation of connective tissue cells	162	2.24E-12	Increased	2.538
Allergy	110	7.57E-09	Increased	0.807
Hypersensitive reaction	111	1.64E-07	Increased	2.023
Inflammation of organ	276	8.78E-11	Decreased	-4.302
Immune response of cells	95	1.07E-10	Increased	5.799
Inflammation of body region	225	3.57E-10	Decreased	-3.950
Inflammatory response	174	6.91E-07	Increased	4.607
Degranulation	60	1.42E-06	Increased	2.341
Fibrosis of tissue	32	1.12E-07	Decreased	-0.663
Metabolism of protein	204	1.45E-06	Increased	3.626
Growth of connective tissue	176	6.17E-13	Increased	3.256
Accumulation of cells	104	5.66E-12	Increased	2.183
Quantity of cells	430	1.06E-18	Increased	6.680

*The differentially expressed gene lists were used as input to calculate functional group enrichment.

VSN and RMA across normal, HPS-1 and HPM samples were used for normalization of the arrays, and limma was used to generate differentially expressed gene analysis with replicate statistics. The overlap in these lists was determined by comparing differentially expressed genes in top ranking criteria using the limma 3.20.9 package output (adj. P-value < 0.05 and FDR < 0.05). Differentially expressed gene lists were initially separately calculated for VSN and RMA, and then lists were compared with each other. When both methods agreed with at least 80%, the differentially expressed gene list based on VSN normalization was used.

## Discussion

In HPS, the melanosomes of melanocytes, dense granules of platelets, and lamellar bodies of type 2 pneumocytes have been studied fairly extensively, but little is known about HuMC granules, which are also LROs. Previously published accounts suggest pulmonary mast cells in patients with fibrotic lung disorders undergo a chronic process of degranulation resulting in altered granule morphology which might relate to the altered granule morphology observed in HPS-1 mast cells (Fig 1E) [23,24]. We thus explored the possibility that abnormalities of the mast cell compartment might exist in patients with Hermansky-Pudlak Syndrome and possible contribute to pathology. Indeed, HPS-1 dermal mast cells, studied *in situ*, contain granules with reproducible ultrastructural abnormalities. HPS-related alterations are also observed in two *in vitro* mast cell models. First, cultured HPS CD34+—derived mast cells display reduced CD117 and FcɛRI expression, possibly contributing to observed differences in degranulation and β-Hex release. HPS-1 HuMCs also exhibit increased expression of the cell surface activation markers CD63 and CD203c [25], and constitutive release of mediators such as histamine and IL-6. The presence of elevated IL-6 in patients’ sera concurrent with elevated IL-6 (and IL-8) in supernatants from nonstimulated HPM cultures (Table 2), which were significantly attenuated by transduction of HPM cells with *HPS1*, suggest that HuMC release of inflammatory cytokines may be one means by which mast cells contribute to pathology in these patients, as seen by numerous activated HuMCs in lung sections. Indeed, serum IL-6 has been shown to be predictive of early functional decline and mortality in interstitial lung disease associated with systemic sclerosis [26].

Imatinib has been used to treat patients whose mast cells lack the D816V mutation [15]. Since HPS-1 HuMCs also lack the D816V mutation, the effect of imatinib was studied by incubating normal control and HPS-1 HuMCs with imatinib and measuring surface markers reflecting apoptosis. Apoptosis was induced after 5 days incubation of both normal and HPS-1 HuMCs with imatinib (Fig 2F), suggesting a possible role for this agent in vivo. Second, the HPM cell line generated from one HPS-1 patient displayed reduced granule formation, impaired β-Hex release, and significant chemotactic ability. Remarkably, the HPM line, which resembles a promastocyte in its growth, ultrastructure, cell surface markers, and secretion of cytokines and proteases, also spontaneously secreted extracellular matrix proteins. Production of the matrix components collagen IV and laminin by mouse mast cells has been reported [27]. HPM cells produce FN-1 and LGALS3 as shown by western blots (Fig 5B) and confocal microscopy (Fig 5C). The detection of LGALS3 is important because this protein is profibrotic and concentrations are significantly higher in HPS-1 pulmonary fibrosis and correlate with disease severity [28]. Functional group analysis of expression changes observed in our datasets confirm enrichment in the functional groups fibrogenesis, differentiation of connective tissue, fibrosis of tissue, growth of connective tissue and degranulation of cells (Table 3). FN-1 and LGALS3 are known to participate in pulmonary fibrosis [28, 29] and be produced by fibroblasts [28] and endothelial cells [30], and now by human HPM cells. The datasets also showed differential upregulation of FN-1, APP, IL-6 and LGALS3, and causal networks constructed from individual relationships curated from the literature have led to the axis FN-1 -> APP -> IL6 as being important (Fig 6C and 6D). Evidence for a relationship between APP and Abeta overproduction and pulmonary fibrosis has been described in Down’s syndrome [31]. Taken together, the presence of the 16-bp duplication (c.1470_1486dup16) in *HPS1* appears to bestow a loss of normal function to the HuMC compartment in patients with *HPS1*.

The newly developed HPM cell line was shown to resemble a promastocyte, and the demonstration in HPM cells of reduced granule formation and β-Hex release, significant chemotaxis and possibly the ability to directly produce matrix components is consistent with this conclusion. Other human mast cell lines such as LAD2, HMC-1 and LUVA cells, are not known to constitutively release cytokines or synthesize matrix components [32]. Initial transfection experiments of HPM cells with normal *HPS1* demonstrated the capability to reduce cell growth rates, restore granulopoiesis, increase granular content of histamine to levels seen with HPS-1 HuMCs. Further, when HPM cells were permanently transduced with HIV-based lentiviral vector and human *HPS1* ORF cDNA lentiviral particles, significant reduction and release of IL-6 and the matrix components FN-1 and LGALS3 are noted, in contrast with IL8 and TNF which are synthesized and regulated differently and not appreciably affected following normal *HPS1* transduction. The observation that LGALS3 is detected in the matrix but not in HPM cell lysate may suggest trafficking, secretion and accumulation of this protein in the matrix compared with cellular levels, which may be comparatively low if the protein is rapidly secreted and deposited extracellularly in the matrix. Since the *HPS1* 16-bp duplication is known to result in defective LRO trafficking, one pathogenetic explanation could be that these molecules are aberrantly trafficked in HPS-1 mast cells, resulting in default secretion.

## Conclusions

Taken together, the *HPS1* gene mutation extends to the mast cell lineage with abnormal granule formation, cell activation, release of cytokines and synthesis of matrix components. More research is needed to better define a role for *HPS1* and confirm our hypothesis that progenitor mast cells in these and similar patients may be involved in pulmonary fibrosis and contribute to patient morbidity, with the possibility of suggesting new approaches to therapy. The HPM cell line may become a useful adjunct to the study of the mechanisms controlling the synthesis of molecules contributing to fibrosis.

## Supporting Information

S1 Fig Pulmonary mast cells in HPS-1 pulmonary fibrosis. Higher magnification view of HPS-1 anti-tryptase stained lung sections. Pulmonary mast cells as seen in Fig 1A are surrounded by reveal red, tryptase positive extracellular granules (smaller arrows); also seen are tryptase positive mast cells (large block arrows), often with a circumferential reddish “blush” representing extracellular tryptase. The scale bar equals 100 microns.
(TIF)

S2 Fig Dermal mast cells in biopsies from normal and HPS-1 patients. Counts and morphology of anti-tryptase stained dermal mast cells showed no differences in numbers or morphology between normal (n = 5) and HPS-1 (n = 6) skin tissue samples.
(TIF)

S3 Fig Ultrastructural images of HuMCs. HuMCs of normal control (upper panels) and HPS-1 patients (lower panels). Granules are labeled according to the classification shown in Fig 1D as follows: a–dense patches, b–cores, c-mottling, d–dense fill, e–less dense fill. The scale bars equal 0.5 microns.
(TIF)

S4 Fig Cytokine release from normal and HPS-1 HuMCs before and following FcɛRI crosslinking. Assays of GM-CSF, TNF-α, TFG-β and PDG2 before and following crosslinking with antigen showed no differences in cytokine levels between normal and HPS-1 HuMCs. Data are from 2 experiments performed in duplicate.
(TIF)

S5 Fig Ultrastructural images of HPM cells. A) HPM cells were immature with a higher nucleus to cytoplasm ratio; B) Fewer, immature granules were noted, and C) Granule content was amorphous with few scroll patterns noted. Scale bars (left to right) equal 2 microns, 500 nm and 100 microns.
(TIF)

S6 Fig Tryptase content of HPM cells. Tryptase quantitation of HPM cells was less than half that of controls and HPS-1 HuMCs, consistent with HPM cell line immaturity and defective granulopoiesis.
(TIF)

S7 Fig PMA and ionomycin release of HPM clones. HPM Clones 3 and 4 showed reduced β-Hex release in the presence of nonspecific stimuli, confirming differences in the exocytic capacity of the cell line that are unrelated to FcɛRI expression.
(TIF)

S8 Fig Serum IL-6 samples from HPS-1 patients and normal controls. Serum IL-6 levels were increased in 4/4 patients with HPS-1 when compared with normal controls. Data are the means + SEM. ***p<0.005. Data from 3 patients is shown in the large graph. The insert shows the highest serum IL-6 level measured (arrow) which was obtained from the one patient with HPS-1 from whom CD34+ cells gave rise to the HPM cell line.
(TIF)

S9 Fig Heatmaps of fibrosis-associated genes COL5A1, LAMA3, FN1, LGALS3 and LGALS3BP. Heatmaps show Collagen type V Alpha 1,2,3 (COL5A1, COL5A2, COL5A3), Laminin Alpha 3 (LAMA3), Fibronectin 1 (FN1), Lectin, Galactoside-Binding, Soluble, 3(LGALS3) and Lectin, Galactoside-Binding, Soluble, 3 Binding Protein (LGALS3BP). Differential expression using multiple probes of COL5A2, LAMA3, FN1 and LGALS3 are shown comparing (A) cultured primary HuMCs from HPS-1 patients (HPS) and normal controls (NORMAL), and (B) HPS1 transduced HPM cells (HPM) and mock transduced HPM cells (CONTROL). Differences in expression regulation for different probes from the same gene may be due to probes recognizing different splicing isoforms. The same gene can upregulate or downregulate its transcripts with different results in the direction of expression. As shown in our pathway analysis (Fig 6C & 6D), the subtle fold change in expression levels for these genes can cause downstream effects that are significant enough to cause changes in pathway enrichment. The fold changes for the probes in these heatmaps are greater than 1.
(TIF)

S1 Video Electron tomography of normal HuMC granules. Normal dermal HuMC granules have increased numbers of granules displaying the dense patch and scroll figure patterns.
(WMV)

S2 Video Electron tomography of HPS-1 HuMC granules. HPS-1 dermal HuMC granules have increased numbers of granules displaying the less-dense fill pattern.
(WMV)

## References

[1] Gahl WA, Huizing M. Hermansky-Pudlak Syndrome In: Gene Reviews, Pagon R.A., Bird T.D., Dolan C.R., Stephens K., Adam M.P., eds. University of Washington, Seattle, WA, 2010;1–29.

[2] Carmona-Rivera C, Simeonov DR, Cardillo ND, Gahl WA, Cadilla CL. A divalent interaction between HPS1 and HPS4 is required for the formation of the biogenesis of lysosome-related organelle complex-3 (BLOC-3). Biochim Biophys Acta. 2013;1833:468–478. 10.1016/j.bbamcr.2012.10.019 23103514 PMC3556189

[3] Ikawa Y, Hess R, Dorward H, Cullinane AR, Huizing M, Gochuico BR, et al In vitro functional correction of Hermansky-Pudlak Syndrome type-1 by lentiviral-mediated gene transfer. Mol Genet Metab. 2015;114:62–65. 10.1016/j.ymgme.2014.11.006 25468649 PMC4279856

[4] Kirshenbaum AS, Goff JP, Semere T, Foster B, Scott LM, Metcalfe DD. Demonstration that human mast cells arise from a progenitor cell population that is CD34+, c-kit+, and expresses aminopeptidase N (CD13). Blood. 1999;4:2333–2342. 10498605

[5] Stone KD, Prussin C, Metcalfe DD. IgE, mast cells basophils, and eosinophils. J Allergy Clin Immunol. 2010;125:S73–80. 10.1016/j.jaci.2009.11.017 20176269 PMC2847274

[6] Cruse G, Bradding P. Mast cells in airway diseases and interstitial lung disease. Eur J Pharmacol. 2015; pii: S0014-2999(15)00408-2. 10.1016/j.ejphar.2015.04.046 PMC4637266 25959386

[7] Huizing M, Helip-Wooley A, Westbroek W, Gunay-Aygun M, Gahl WA. Disorders of lysosome-related organelle biogenesis: clinical and molecular genetics. Annu Rev Genomics Hum Genet. 2008;9:359–386. 10.1146/annurev.genom.9.081307.164303 18544035 PMC2755194

[8] Gahl WA, Brantly M, Kaiser-Kupfer MI, Iwata F, Hazelwood S, Shotelersuk V, et al Genetic defects and clinical characteristics of patients with a form of oculocutaneous albinism (Hermansky-Pudlak syndrome). N Engl J Med. 1998;338:1258–1264. 9562579 10.1056/NEJM199804303381803

[9] Bandara G, Metcalfe DD, Kirshenbaum AS. Growth of human mast cells from bone marrow and peripheral blood-derived CD34+ pluripotential hematopoietic cells In Methods in Molecular Biology, vol. 1220, Hughes MR, McNagny KM (eds.), Humana Press, NY, 2015;155–162. 25388250 10.1007/978-1-4939-1568-2_10 PMC6396975

[10] Brockow K, Akin C, Huber M, Scott LM, Schwartz LB, Metcalfe DD. Levels of mast-cell growth factors in plasma and in suction skin blister fluid in adults with mastocytosis: correlation with dermal mast cell numbers and mast cell tryptase. J Allergy Clin Immunol. 2002;109:82–88. 11799370 10.1067/mai.2002.120524

[11] Chan EC, Bai Y, Kirshenbaum AS, Fischer ER, Simakova O, Bandara G et al Mastocytosis associated with a rare germline KIT K509I mutation displays a well-differentiated mast cell phenotype. J Allergy Clin Immunol. 2014;134:178–187. 10.1016/j.jaci.2013.12.1090 24582309 PMC4125511

[12] Ali K, Bilancio A, Thomas M, Pearce W, Gilfillan AM, Tkaczyk C, et al Essential role for the p110delta phosphoinositide 3-kinase in the allergic response. Nature. 2004;431:1007–1011. 15496927 10.1038/nature02991

[13] Kirshenbaum AS, Swindle E, Kulka M, Wu Y, Metcalfe DD. Effect of lipopolysaccharide (LPS) and peptidoglycan (PGN) on human mast cell numbers, cytokine production, and protease composition. BMC Immunol. 2008;9: 45–52. 10.1186/1471-2172-9-45 18687131 PMC2527549

[14] Kuehn H-S, Radinger M, Gilfillan AM. Measuring mast cell mediator release. Curr Protoc Immunol. 2010; 91:7.38.1–7.38.9. 10.1002/0471142735.im0738s91 PMC2982193 21053305

[15] Lahortiga I, Akin C, Cools J, Wilson TM, Mentens N, Arthur DC, et al Activity of imatinib in systemic mastocytosis with chronic basophilic leukemia and a PRKG2- PDGFRB fusion. Haematologica. 2008;193:49–56. 10.3324/haematol.11836 18166785

[16] Hazelwood S, Shotelersuk V, Wildenberg SC, Chen D, Iwata F, Kaiser-Kupfer MI, Evidence for locus heterogeneity in Puerto Ricans with Hermansky-Pudlak syndrome. Am J Hum Genet. 1997; 61:1088–1094. 9345105 10.1086/301611 PMC1716022

[17] Cruse G, Beaven MA, Ashmole I, Bradding P, Gilfillan AM, Metcalfe DD. A truncated splice-variant of the FcεRIβ receptor subunit is critical for microtubule formation and degranulation in mast cells. Immunity. 2013; 38, 906–917. 10.1016/j.immuni.2013.04.007 23643722 PMC3694348

[18] Tkaczyk C. Metcalfe DD, Gilfillan AM. Determination of protein phosphorylation in FcɛRI-activated human mast cells by immunoblot analysis requires protein extraction under denaturing conditions. J Immunol Methods. 2002;268:239–243. 12215392 10.1016/s0022-1759(02)00210-7

[19] Dvorak AM. Degranulation and recovery from degranulation of basophils and mast cells. Chem Immunol Allergy. 2005;85:205–251. 15970659 10.1159/000086519

[20] Steinestel K, Wardelmann E, Hartmann W, Grunewald I. Regulators of actin dynamics in gastrointestinal tract tumors. Gastroenterology Research and Practice. 2015; 10.1155/2015/930157 PMC4539459 26345720

[21] Bildyug N, Bozhokina E, Khaitlina S. Contribution of a-smooth muscle actin and extracellular matrix to the in vitro reorganization of cardiomyocyte contractile system. Cell Biol Int 9999 (2015) 1–6. 10.1002/cbin.10577 26732641

[22] Walther CG, Whitfield R, James DC. Importance of interaction between integrin and actin cytoskeleton in suspension adaptation of CHO cells. Appl Biochem Biotechnol. 10.1007/s12010-015-1945-z PMC4858566 26679704

[23] Kawanami O, Ferrans VJ, Fulmer JD, Crystal RG. Ultrastructure of pulmonary mast cells in patients with fibrotic lung disorders. Lab Invest. 1979;40:717–734. 449278

[24] Dvorak AM, Schleimer RP, Schulman ES, Lichtenstein LM. Human mast cells use conservation and condensation mechanisms during recovery from degranulation. In vitro studies with mast cells purified from human lungs. Lab Invest. 1986;54:663–678. 2423778

[25] MacGlashan D. Marked differences in the signaling requirements for expression of CD203c and CD11b versus CD63 expression and histamine release in human basophils. Int Arch Allergy Immunol. 2012;159:243–252. 10.1159/000332150 22722613 PMC3533433

[26] De Lauretis A, Sestini P, Pantelidis P, Hoyles R, Hansell DM, Goh NS, et al Serum interleukin 6 is predictive of early functional decline and mortality in interstitial lung disease associated with systemic sclerosis. J Rheumatol. 2013;40:435–446. 10.3899/jrheum.120725 23378460

[27] Thompson HL, Burbelo PD, Gabriel G, Yamada Y, Metcalfe DD. Murine mast cells synthesize basement membrane components. A potential role in early fibrosis. J Clin Invest.1991;87:619–623. 1991845 10.1172/JCI115038 PMC296351

[28] Cullinane AR, Yeager C, Dorward H, Carmona-Rivera C, Wu HP, Moss J, et al Dysregulation of Galectin-3. Am J Respir Cell Mol Biol. 2014;50:605–613. 10.1165/rcmb.2013-0025OC 24134621 PMC4068929

[29] Lu P, Takai K, Weaver VM, Werb Z. Extracellular matrix degradation and remodeling in development and disease. Cold Spring Harb Perspect Biol. 2011;3:1–24. 10.1101/cshperspect.a005058 PMC3225943 21917992

[30] Kusuma S, Zhao S, Gerecht S. The extracellular matrix is a novel attribute of endothelial progenitors and of hypoxic mature endothelial cells. FASEB J. 2012;26,4925–4936. 10.1096/fj.12-209296 22919069 PMC3509053

[31] Moritake H, Yamada A, Kimoto Y, Sawa D, Shimonodan H, Nunoi H. Acute megakaryoblastic leukemia and severe pulmonary fibrosis in a child with Down syndrome: successful treatment with ultra low-dose cytarabine using GATA1 mutation to monitor minimal residual disease. Am J Hematol. 2012;87:447–450. 10.1002/ajh.23130 22389016

[32] Kirshenbaum AS, Petrik A, Walsh R, Kirby TL, Vepa S, Wangsa D, et al A Ten Year Retrospective Analysis of the Distribution, Use and Phenotypic Characteristics of the LAD2 Human Mast Cell Line. Int Arch Allergy Immunol. 2014;164:265–270. 10.1159/000365729 25195635 PMC4201868

