# Supplementary figures and images for: Immunophenotypic and Ultrastructural Analysis of Mast Cells in Hermansky-Pudlak Syndrome Type-1: A Possible Connection to Pulmonary Fibrosis
Source: PLoS One. 2016 Jul 26;11(7):e0159177. doi: 10.1371/journal.pone.0159177 (PMC4961407; doi:10.1371/journal.pone.0159177)

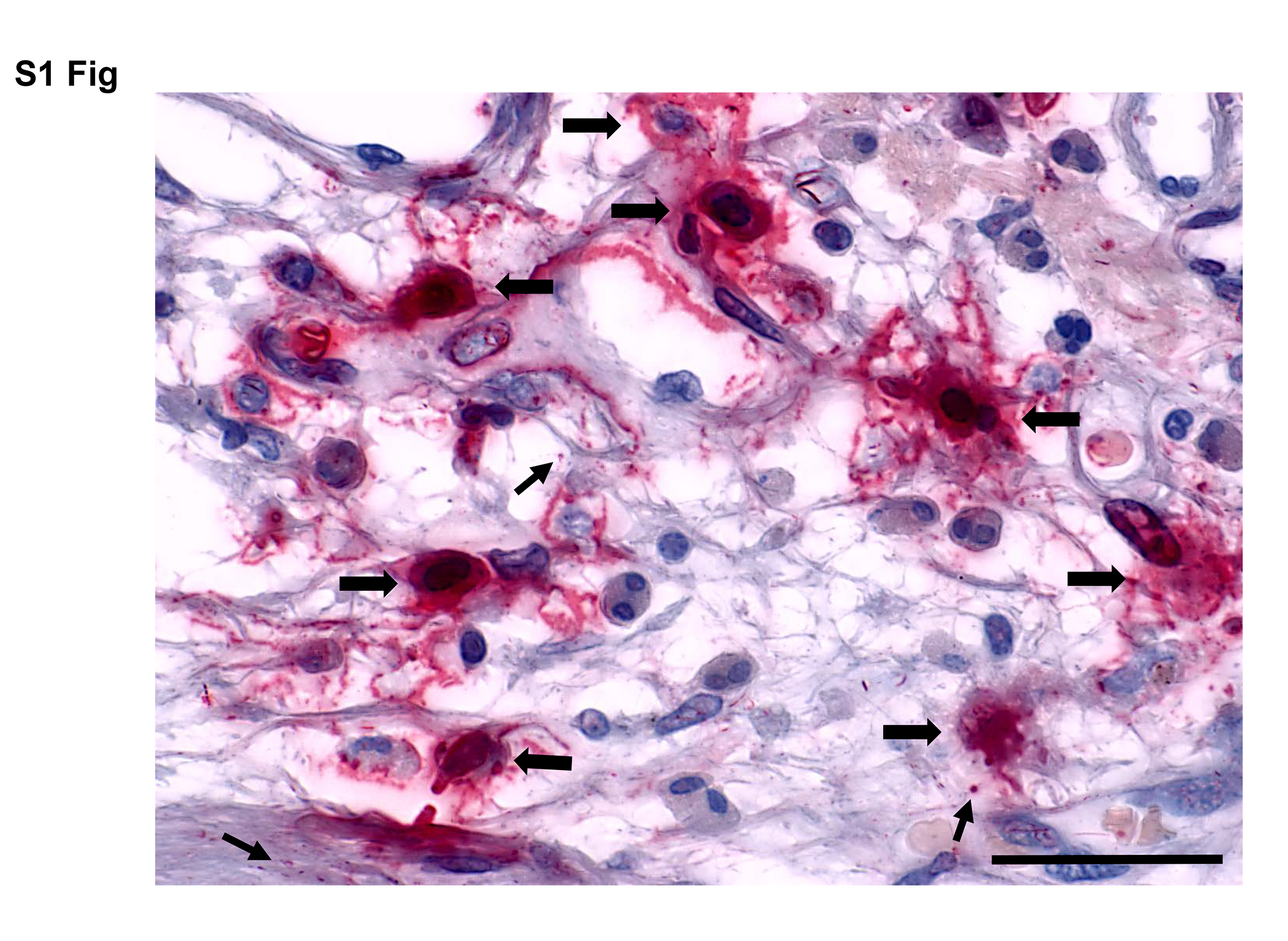

Supplement: S1 Fig — Higher magnification view of HPS-1 anti-tryptase stained lung sections. Pulmonary mast cells as seen in Fig 1A are surrounded by reveal red, tryptase positive extracellular granules (smaller arrows); also seen are tryptase positive mast cells (large block arrows), often with a circumferential reddish “blush” representing extracellular tryptase. The scale bar equals 100 microns. (TIF) [file pone.0159177.s001.tif]

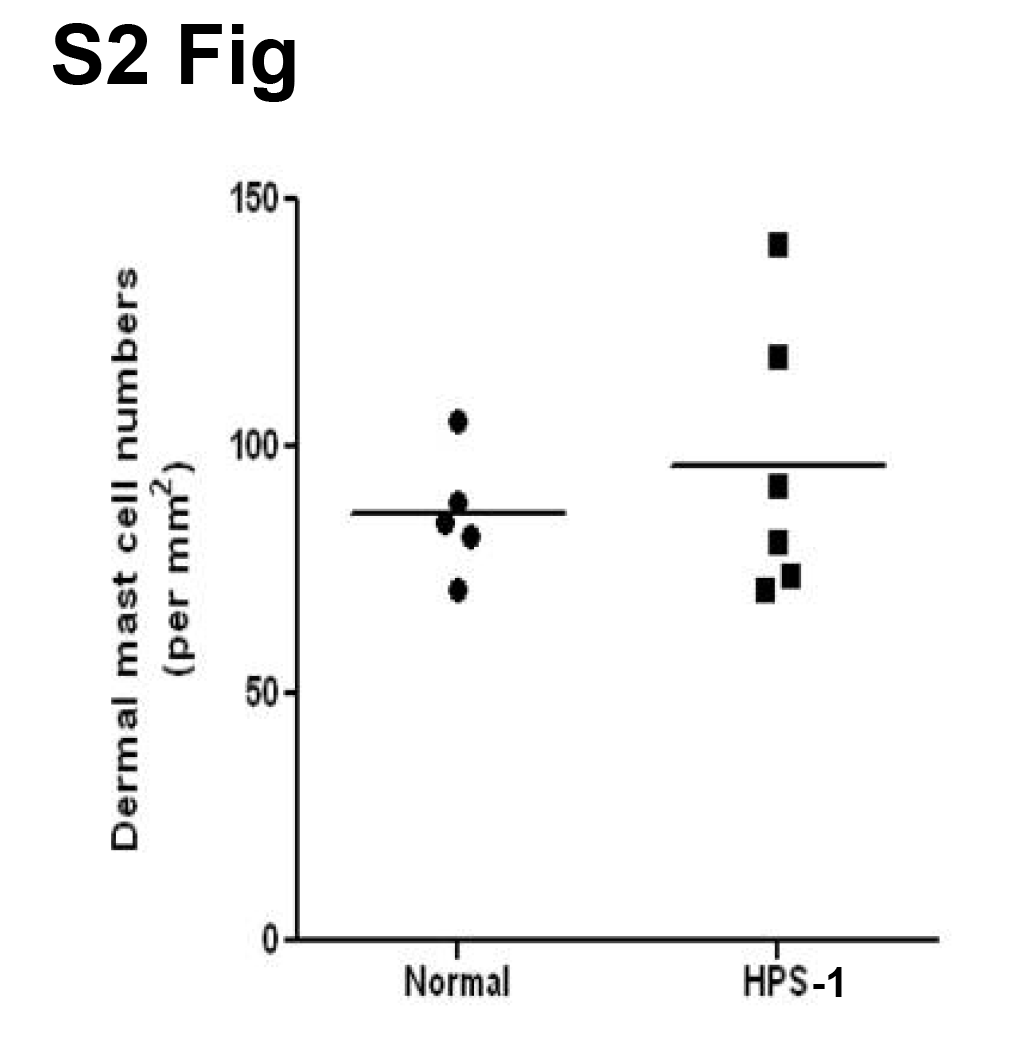

Supplement: S2 Fig — Counts and morphology of anti-tryptase stained dermal mast cells showed no differences in numbers or morphology between normal (n = 5) and HPS-1 (n = 6) skin tissue samples. (TIF) [file pone.0159177.s002.tif]

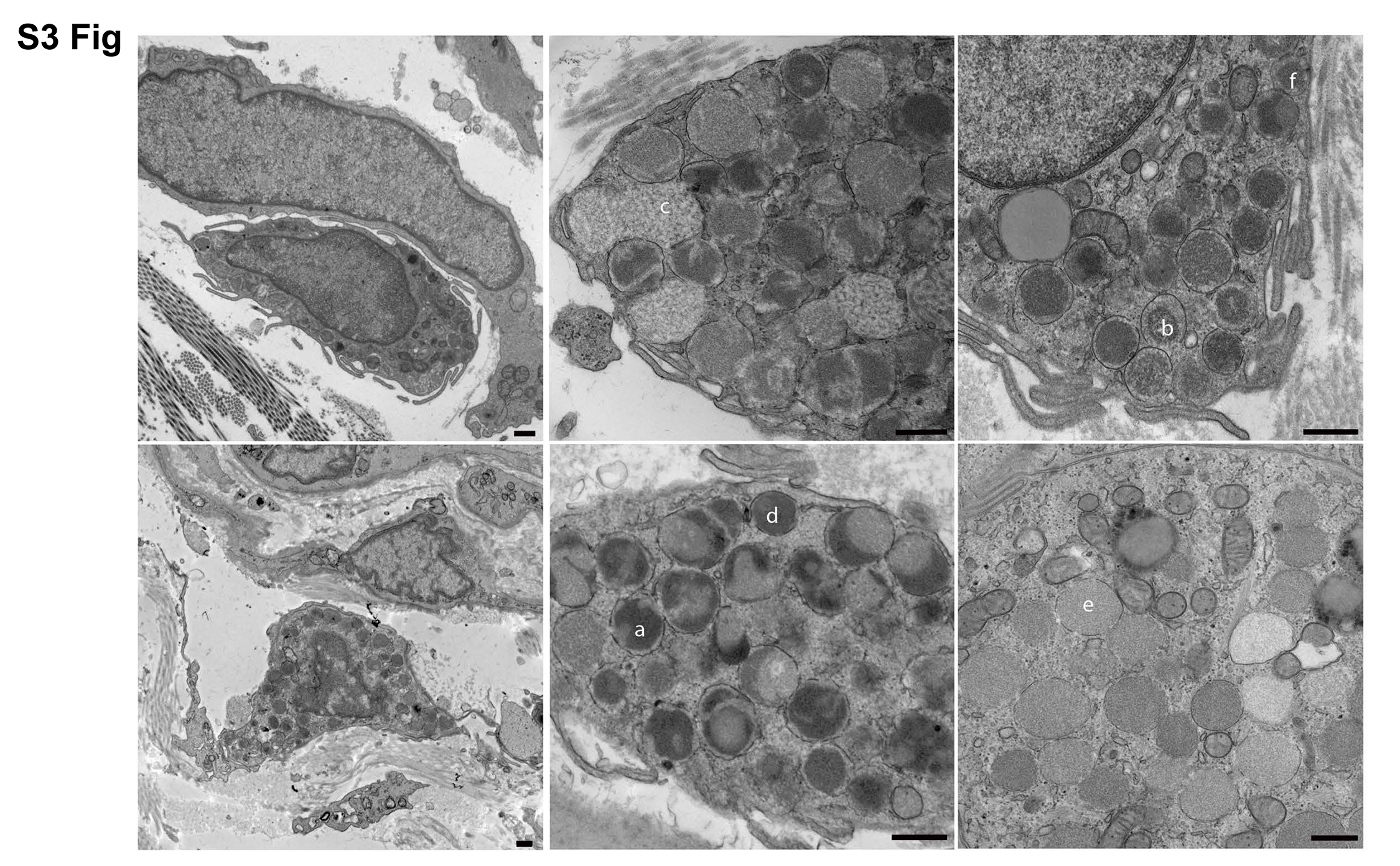

Supplement: S3 Fig — HuMCs of normal control (upper panels) and HPS-1 patients (lower panels). Granules are labeled according to the classification shown in Fig 1D as follows: a–dense patches, b–cores, c-mottling, d–dense fill, e–less dense fill. The scale bars equal 0.5 microns. (TIF) [file pone.0159177.s003.tif]

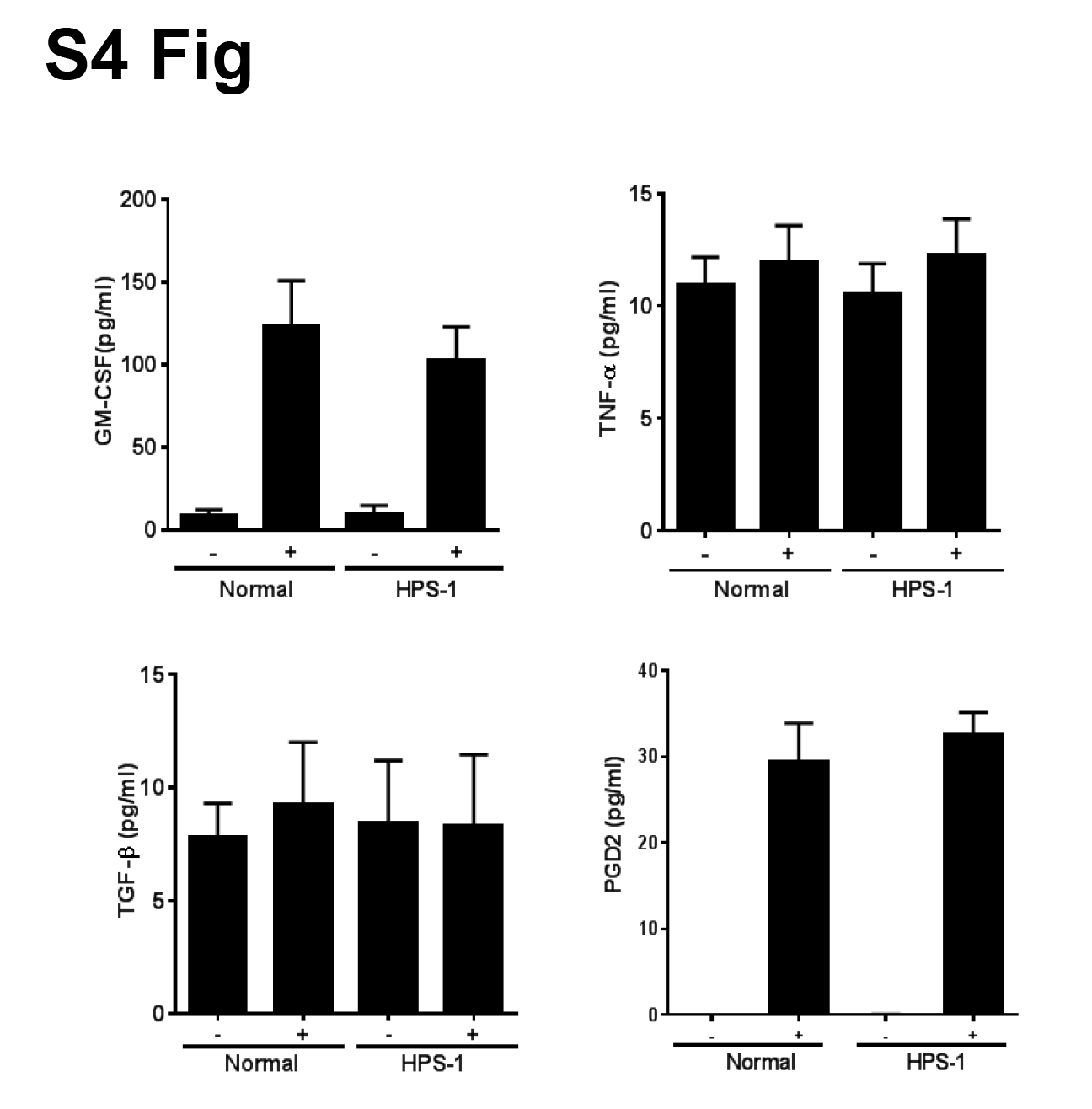

Supplement: S4 Fig — Assays of GM-CSF, TNF-α, TFG-β and PDG2 before and following crosslinking with antigen showed no differences in cytokine levels between normal and HPS-1 HuMCs. Data are from 2 experiments performed in duplicate. (TIF) [file pone.0159177.s004.tif]

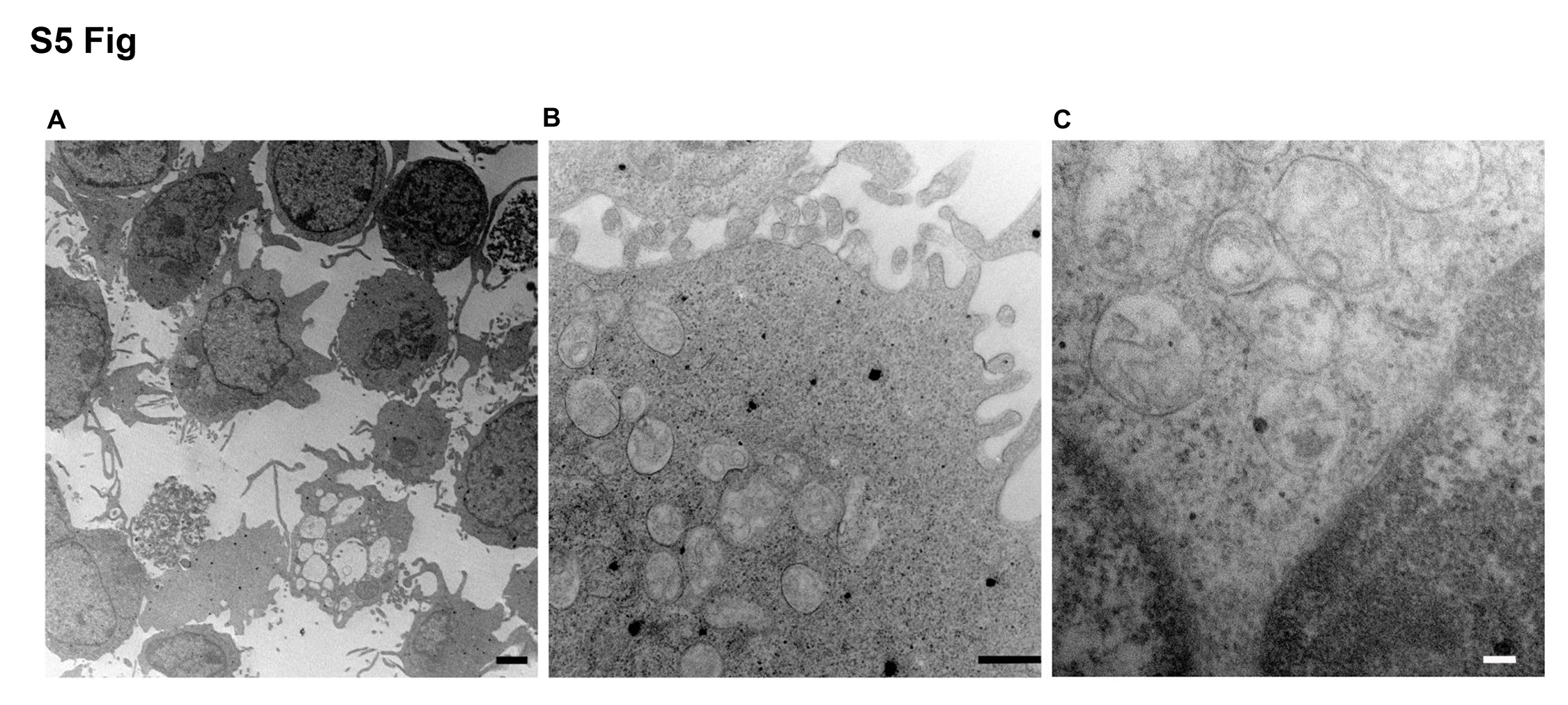

Supplement: S5 Fig — A) HPM cells were immature with a higher nucleus to cytoplasm ratio; B) Fewer, immature granules were noted, and C) Granule content was amorphous with few scroll patterns noted. Scale bars (left to right) equal 2 microns, 500 nm and 100 microns. (TIF) [file pone.0159177.s005.tif]

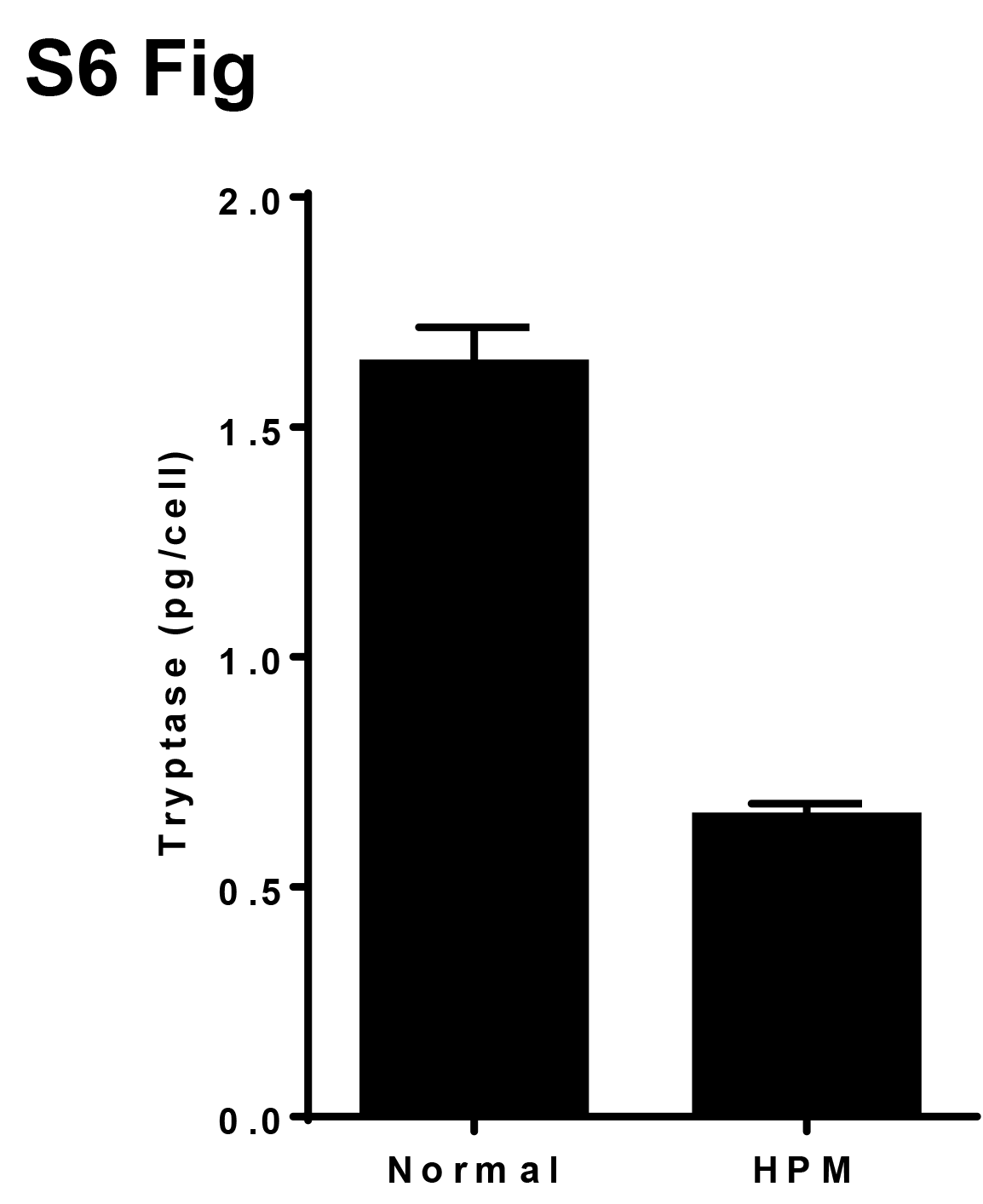

Supplement: S6 Fig — Tryptase quantitation of HPM cells was less than half that of controls and HPS-1 HuMCs, consistent with HPM cell line immaturity and defective granulopoiesis. (TIF) [file pone.0159177.s006.tif]

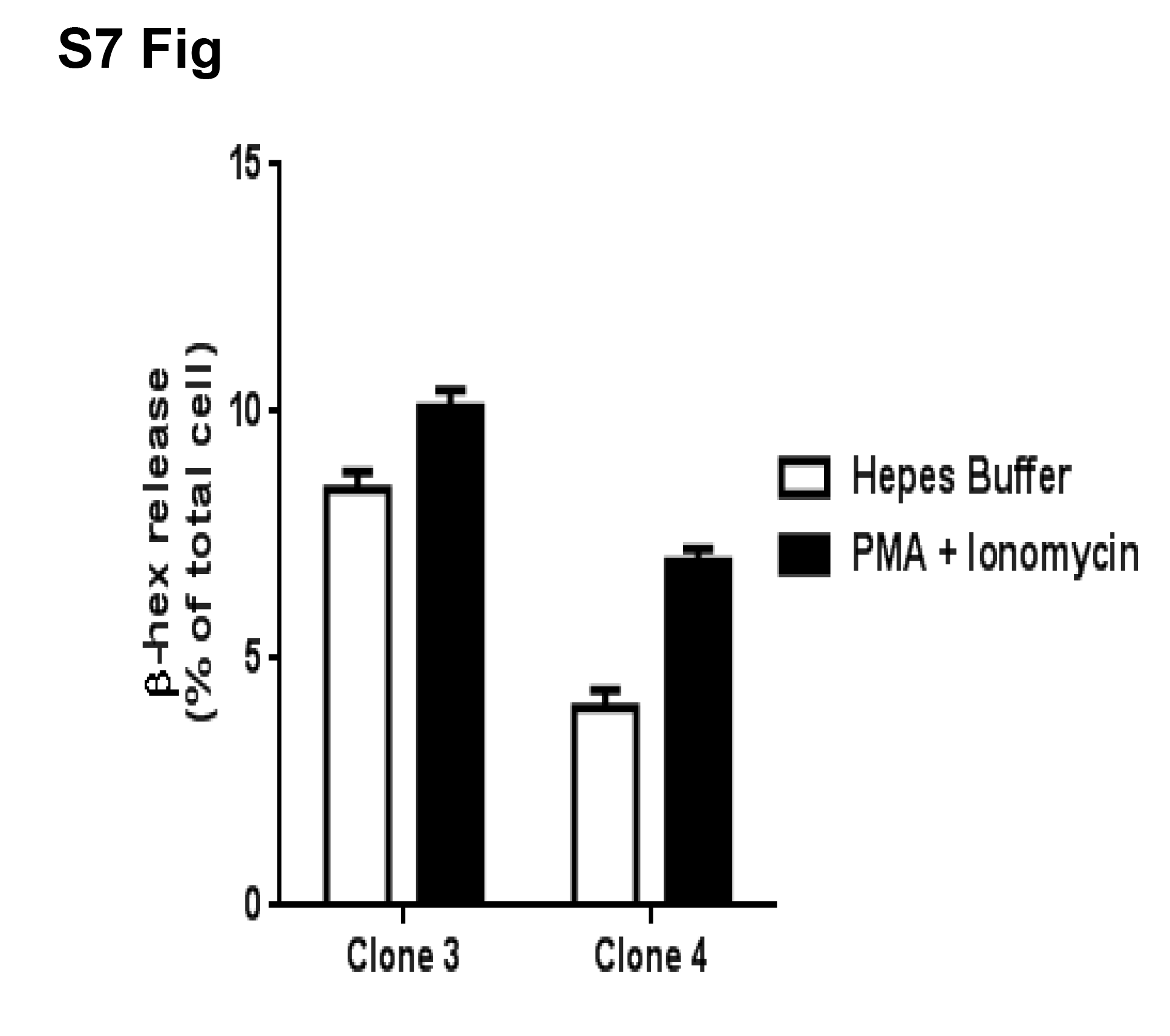

Supplement: S7 Fig — HPM Clones 3 and 4 showed reduced β-Hex release in the presence of nonspecific stimuli, confirming differences in the exocytic capacity of the cell line that are unrelated to FcɛRI expression. (TIF) [file pone.0159177.s007.tif]

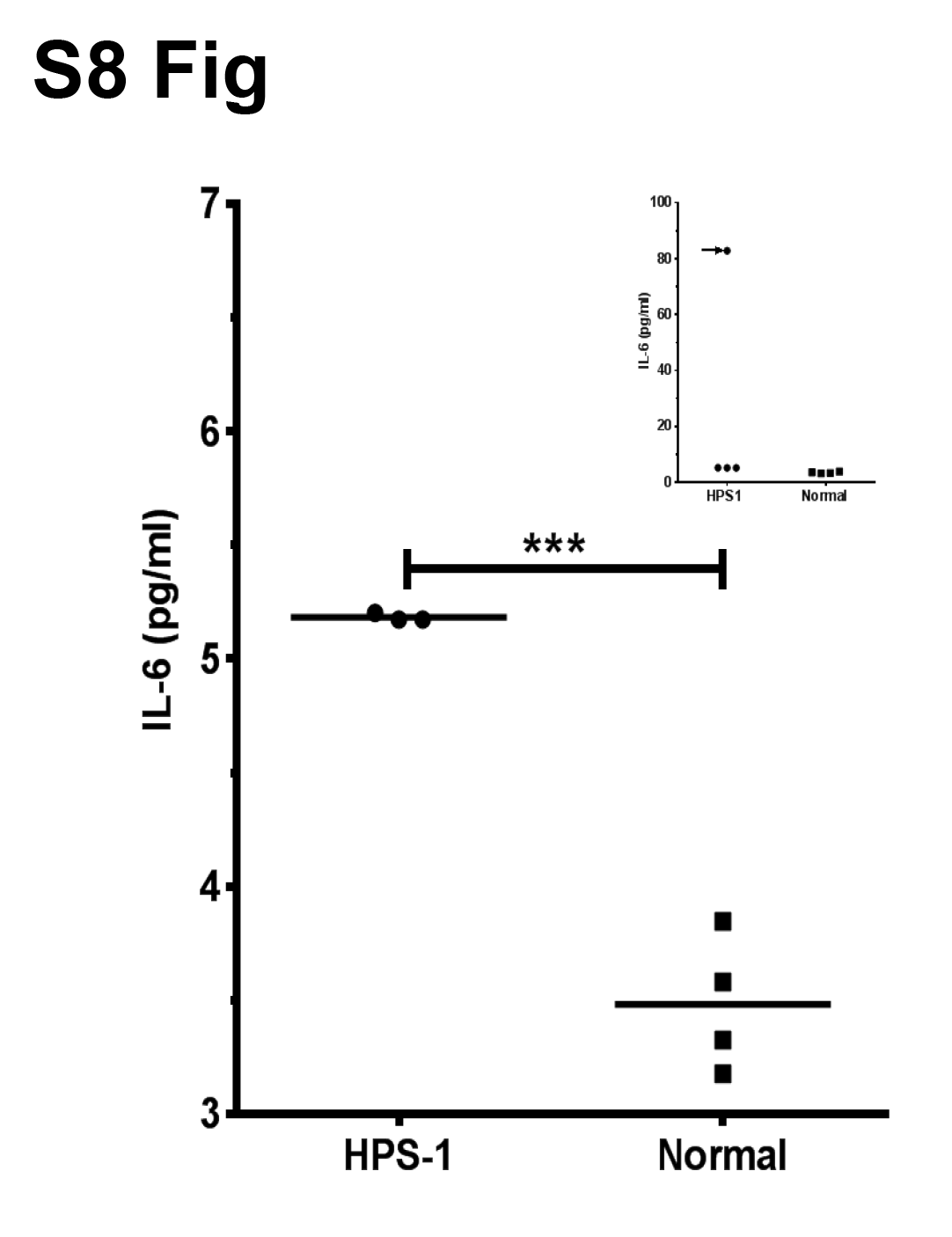

Supplement: S8 Fig — Serum IL-6 levels were increased in 4/4 patients with HPS-1 when compared with normal controls. Data are the means + SEM. ***p<0.005. Data from 3 patients is shown in the large graph. The insert shows the highest serum IL-6 level measured (arrow) which was obtained from the one patient with HPS-1 from whom CD34+ cells gave rise to the HPM cell line. (TIF) [file pone.0159177.s008.tif]

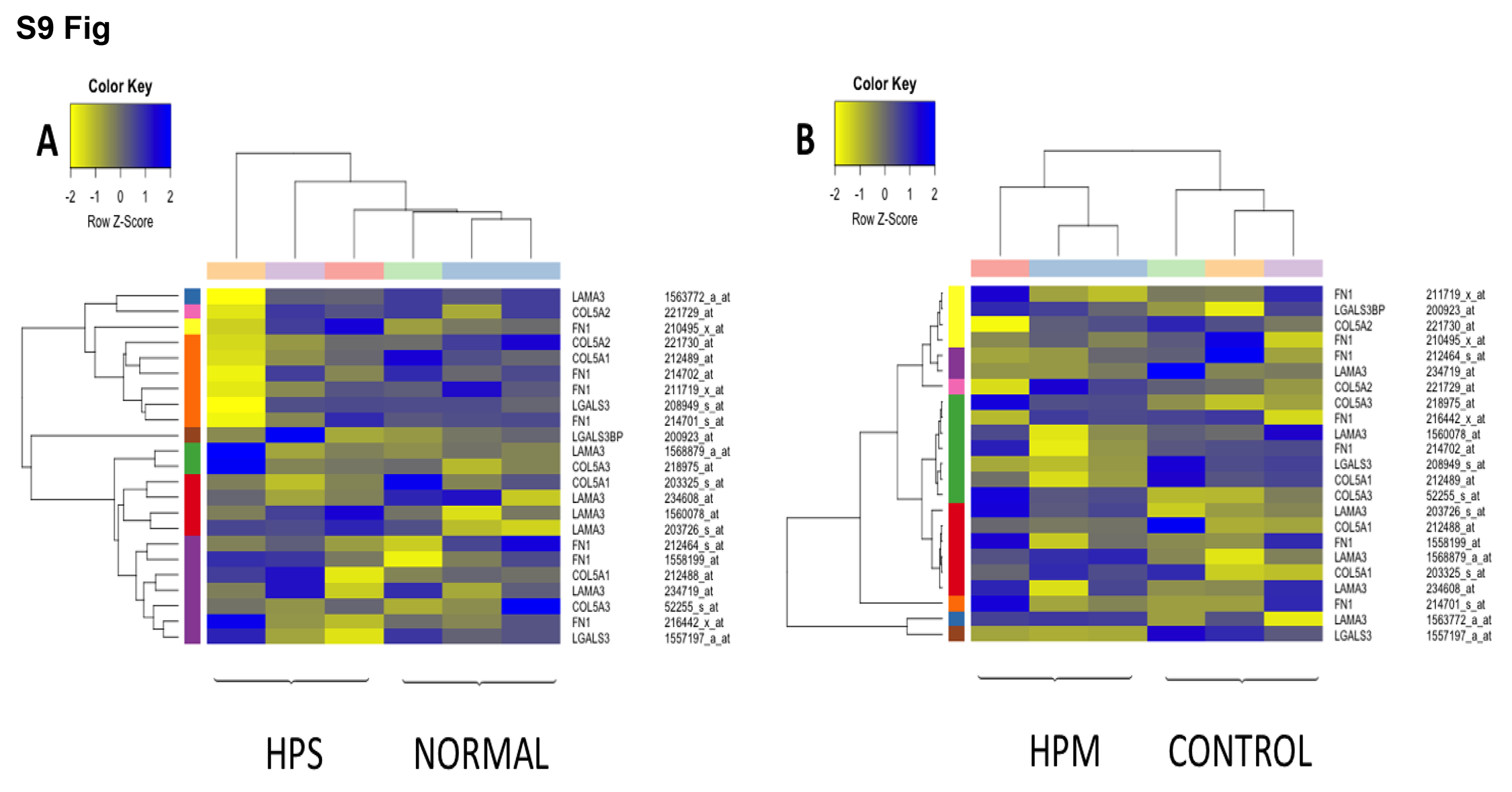

Supplement: S9 Fig — Heatmaps show Collagen type V Alpha 1,2,3 (COL5A1, COL5A2, COL5A3), Laminin Alpha 3 (LAMA3), Fibronectin 1 (FN1), Lectin, Galactoside-Binding, Soluble, 3(LGALS3) and Lectin, Galactoside-Binding, Soluble, 3 Binding Protein (LGALS3BP). Differential expression using multiple probes of COL5A2, LAMA3, FN1 and LGALS3 are shown comparing (A) cultured primary HuMCs from HPS-1 patients (HPS) and normal controls (NORMAL), and (B) HPS1 transduced HPM cells (HPM) and mock transduced HPM cells (CONTROL). Differences in expression regulation for different probes from the same gene may be due to probes recognizing different splicing isoforms. The same gene can upregulate or downregulate its transcripts with different results in the direction of expression. As shown in our pathway analysis (Fig 6C & 6D), the subtle fold change in expression levels for these genes can cause downstream effects that are significant enough to cause changes in pathway enrichment. The fold changes for the probes in these heatmaps are greater than 1. (TIF) [file pone.0159177.s009.tif]
